# Multivalent mpox protein nanoparticle vaccines confer cross-protection against orthopoxvirus infection

**DOI:** 10.1371/journal.ppat.1013389

**Published:** 2025-08-21

**Authors:** Lijun Zhang, Chuanyu Liu, Chenxi Yang, Xueqi Xiao, Senyu Xu, Shengfeng Wan, Kun Xu, Yan Li, Lianpan Dai

**Affiliations:** 1 Laboratory of Pathogenic Microbiology and Immunology, Institute of Microbiology, Chinese Academy of Sciences, Beijing, China; 2 Xiangyang Central Hospital, Affiliated Hospital of Hubei University of Arts and Science, Xiangyang, Hubei Province, China; 3 University of Chinese Academy of Sciences, Beijing, China; 4 Medical School, University of Chinese Academy of Sciences, Beijing, China; 5 Research Network of Immunity and Health (RNIH), Beijing Institutes of Life Science, Chinese Academy of Sciences, Beijing, China; State University of New York Upstate Medical University, UNITED STATES OF AMERICA

## Abstract

The outbreak of mpox since 2022 has driven the development of mpox virus (MPXV)-specific, subunit-based, next-generation vaccines, instead of the currently used live-attenuated vaccinia virus (VACV) vaccines. Here, we describe a self-assembling protein nanoparticle against MPXV using lumazine synthase to present viral surface proteins. Multivalent nanoparticles elicited broader and stronger immune responses against MPXV and provided superior heterologous protection in rodent models against lethal VACV challenges compared to monovalent formulations. The three antigens with the best protective efficacy (intracellular mature virus antigens M1 and E8, and extracellular enveloped virus antigen B6) were further combined as the trivalent cocktail or mosaic nanoparticle. The trivalent nanoparticles elicited higher humoral responses compared to the modified vaccinia virus Ankara, and were protective against lethal VACV challenge in mice, with the protection correlation revealed. These findings highlight the potential of multivalent nanoparticle as vaccines against MPXV and other orthopoxviruses.

## Author summary

The global mpox outbreak in 2022 highlighted the urgent need for safer and more effective alternatives to traditional live-attenuated smallpox vaccines. In response, we engineered a novel vaccine employing self-assembling nanoparticles to display three essential mpox viral proteins-M1, E8, and B6. This trivalent nanoparticle vaccine elicited significantly stronger and broader immune responses in animal models. Importantly, mice immunized with our vaccine achieved complete protection against lethal challenges with related orthopoxviruses, surpassing both individual protein vaccines and traditional live-attenuated smallpox vaccines. By eliminating the safety concerns associated with live-virus vaccines and delivering robust, multi-target immune responses, our approach represents a promising advancement in vaccine development. These findings lay a critical foundation for safer, highly effective vaccines against mpox virus and other orthopoxviruses, offering valuable preparedness for future outbreaks.

## Introduction

Mpox is a zoonotic viral disease caused by the mpox virus (MPXV), showing clinical symptoms resembling smallpox [1], such as fever, lymphadenopathy, and rash [2]. Since May 2022, mpox outbreaks have resulted in 99,176 confirmed cases and 208 deaths across 116 countries (who.int) [3–5], prompting the World Health Organization (WHO) to declare it a Public Health Emergency of International Concern (PHEIC) in July 2022 [6]. Although the PHEIC was lifted following a decline in cases [7], a resurgence of mpox infections in the Democratic Republic of Congo (DRC) in 2023 underscored ongoing challenges. Notably, while the 2022 epidemic was dominated by the less virulent clade II strain, the more lethal clade I strain, with a mortality rate exceeding 10%, emerged in the DRC in 2023 and spread to neighboring countries by 2024 [8,9], leading the WHO to declare a second global health emergency [10,11]. As of 31 December 2024, the African had 23,489 confirmed cases, while the global total of 12,4753 infected people cover 128 countries had been reported by WHO [12]. These outbreaks highlight the critical need for targeted vaccination strategies, particularly to control clade Ib strains with the pandemic potential.

MPXV belongs to the orthopoxviruses genus, which includes variola virus, the causative agent of smallpox, and vaccinia virus (VACV) [1]. Orthopoxviruses have double-stranded DNA genomes with high antigen similarity, enabling cross-protection among them [13,14]. The eradication of smallpox was achieved by leveraging this cross-protection through global immunization with VACV as a live vaccine [15]. Although no antiviral drug has been approved specifically for mpox, several smallpox-licensed drugs including-tecovirimat, brincidofovir and cidofovir-have been used as Pre-Exposure Prophylaxis, off-label, and under Emergency Use Authorization during recent mpox outbreaks [16]. Consequently, an effective vaccine should be the primary preventive measure for potential mpox outbreak. So far, the U.S. Food and Drug Administration (FDA) has conditionally approved three smallpox live attenuated vaccines-JYNNEOS [17,18], LC16 and OrthopoxVac-for vaccination against MPXV [19]. JYNNEOS is a modified vaccinia virus-based live, non-replicating vaccine (MVA-BN), and its effectiveness against diagnosed mpox in individuals receiving a complete two-dose regimen was estimated at 66% during the 2022 outbreaks [20,21]. LC16 is approved for pediatric use [22,23], while OrthopoxVac has shown limited protective efficacy [24]. Despite of these options, the development of vaccines directly targeting MPXV remains critical.

MPXV, like other orthopoxviruses, has two distinct infectious forms, the intracellular mature virion (IMV) and extracellular enveloped virion (EEV) [25,26]. Each viral form has distinct surface antigens that facilitate the attachment of IMVs to cells (A29, E8 and H3), viral assembly and entry (M1), and viral transmission of EEVs (A35 and B6) [27–30]. While immune responses elicited by a single antigen can provide some protection from infection, study involving vaccines and monoclonal antibody prophylaxis in animals has shown the benefit of combining antibodies to multiple EEV and IMV antigens [27,28,31–36]. Notably, nanoparticle vaccine platforms have emerged as a promising strategy for presenting multiple antigns [37], as demonstrated in vaccines for Covid-19 [38], influenza [39], and hepatitis B [40]. These platforms effectively enhance immune responses by priming antigen-presenting cells like dendritic cells and macrophages in the lymph nodes of the host, leading to enhanced B cell responses and improved broad and long-term immunity [38–42].

Here, we describe the development of a multivalent nanoparticle vaccine targeting MPXV using a 60-mer lumazine synthase (LuS) protein covalently linked with IMV and EEV antigens via a SpyTag/SpyCatcher (ST/SC) system which facilitate conjugation of target antigens to nanoparticle under physiological conditions [42,43]. Compared to monovalent ones, multivalent nanoparticles demonstrated better cross-protection against a genetically related VACV challenge in mice, with significantly higher immunogenicity. Furthermore, our trivalent nanoparticle vaccines- containing three antigens (IMV proteins M1 and E8, EEV protein B6) demonstrated robust immunogenicity and significant cross-protection against VACV in mice. These findings advance our understanding of neutralizing antibody titers correlation with orthopoxviruses viral loads in mice tissues and offer a promising approach for further mpox vaccine development.

## Results

### Generation and characterization of MPXV nanoparticle

**Fig 1:**
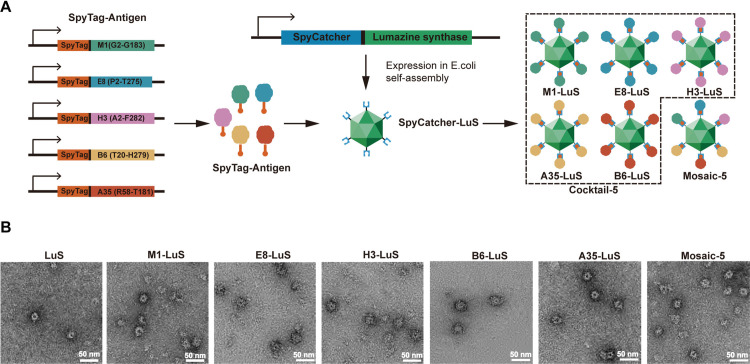
Design and characterization of MPXV nanoparticle vaccines. (A) Schematic diagram illustrating the construct design and production of the SpyTag-antigens including M1, E8, H3, B6, A35, and SpyCatcher-LuS. (B) Negative-staining electron microscopy of each antigen-LuS and mosaic-5 nanoparticles, and observed under Tecnai Spirit microscope 120 kV (scale bar = 50 nm).

We selected five surface proteins from MPXV clade I (Zaire-96 strain, accession no. AF380138.1) known to elicit neutralizing antibodies as the immunogens, three from IMV (M1, E8 and H3) and two from EEV (A35 and B6) [32,35,44,45] which share high amino acid identity with VACV antigens (98.4%, 91.7%, 94.8%, 97%, and 98.1% amino acid identity,S1 Fig). The ectodomain of these five MPXV proteins, expressed in Expi293F cells, were presented by a 60-mer, self-assembling lumazine (LuS) nanoparticle, expressed in *E.coli*, via the ST/SC covalent coupling (Fig 1A). Monovalent nanoparticles M1-LuS, E8-LuS, H3-LuS, B6-LuS, and A35-LuS were produced separately and combined as pentavalent cocktail (namely cocktail-5). Mosaic pentavalent nanoparticle (namely mosaic-5) was produced by displaying these five SpyTagged antigens on a LuS simultaneously (Fig 1A). The individual SpyTagged antigens, and the protein sizes before and after ST/SC coupling were confirmed by analytical gel filtration and gel electrophoresis (S2 Fig). The antigen-decorated nanoparticles were further confirmed by negative staining electron microscopy (Fig 1B). Furthermore, we used specific mAbs to detect each antigen presented on the mosaic nanoparticle using a biolayer interferometry assay (BLI), and confirmed the display of all five antigens (S3 Fig). These results suggested successful presentation of MPXV antigens on the LuS particle surface.

### Immunogenicity of MPXV nanoparticle vaccines in mice

**Fig 2:**
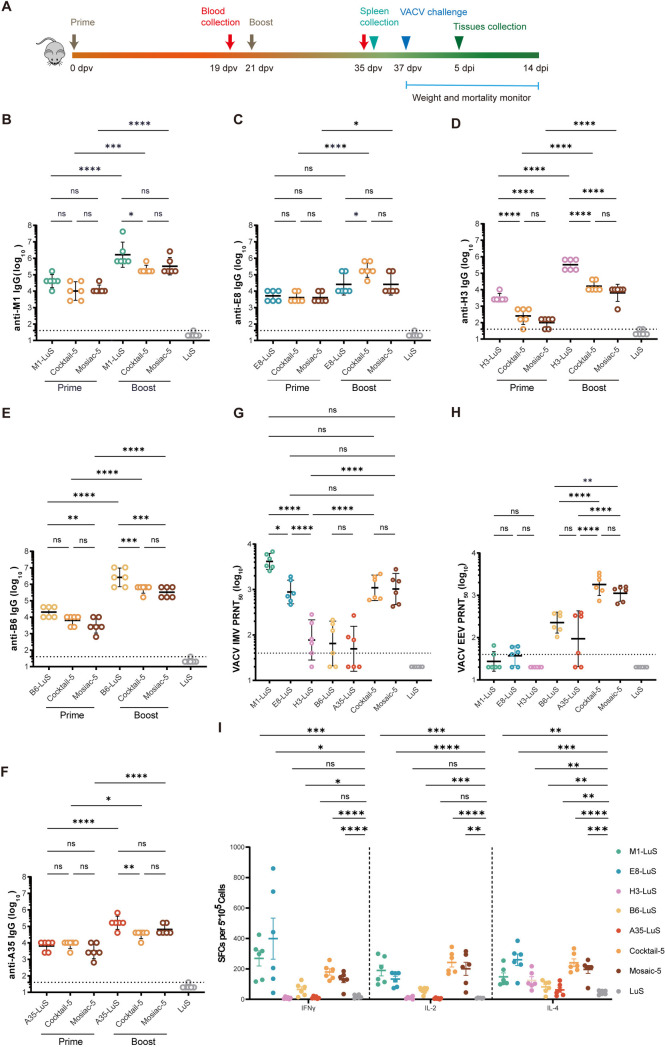
MPXV nanoparticle vaccines elicit humoral and cellular immune responses. (A) Vaccination scheme for mice immunization. Mice (n = 12) of each group were immunized subcutaneously (s.c) with two doses (2 μg/dose) of MPXV nanoparticle vaccines (M1-LuS, E8-LuS, H3-LuS, B6-LuS, A35-LuS, cocktail-5 or mosaic-5) or with empty particles LuS at a 21-day interval, and were challenged with VACV-WR at 37 dpv. Mouse icon adapted from an OpenClipart public-domain image (SVG ID 22277). (B-F) Anti-specific IgG titers for MPXV antigens, including M1 (B), E8 (C), H3 (D), B6 (E), and A35 (F) were measured by ELISA in sera collected from mice (n = 6) at 19 and 35 dpv. (G-H) Neutralizing activities of sera (n = 6) against IMV (G) and EEV (H) were determined by plaque reduction neutralization (PRNT) assay against VACV-WR infection, and the 50% plaque reduction neutralization (PRNT_50_) was calculated. The data of (B-H) are geometric mean titer (GMT) with a 95% confidence interval (CI). (I) Cellular immune response of mice (n = 6) after vaccination with two doses of vaccine (2 μg/dose) was measured by ELISpot assay with 2.5 × 10⁵ splenocytes per well. Secretion levels of IFNγ, IL-2, and IL-4 by splenocytes after stimulation with overlapping peptide pools or a mixture of MPXV antigens M1, E8, H3, B6, and A35 were detected to evaluate cellular immune responses. The data of (I) means ± SEM. *P* values were determined with one-way ANOVA with multiple comparisons test. (ns, *P* > 0.05; *, *P* < 0.05; **, *P* < 0.01; ***, *P* < 0.001; ****, *P* < 0.0001). The horizontal dashed line indicates the lower limit of detection (LLOD).

To assess the immunogenicity of MPXV nanoparticle vaccine candidates, BALB/c mice were immunized subcutaneously (s.c.) with two doses of 2 µg per dose of monovalent nanoparticles, cocktail-5 and mosaic-5, three weeks apart, adjuvanted with AddaVax (a squalene-based oil-in-water adjuvant) (Fig 2A). Empty nanoparticle (LuS) plus adjuvant was given as the sham control.

For humoral response, serum samples were collected at 19- and 35-days post-vaccination (dpv) and specific IgG antibody titers were quantified by ELISA. Both cocktail-5 and mosaic-5 vaccines induced robust antibody responses to each antigen, although not superior to those of the monovalent nanoparticles (Fig 2B-2F). Serum samples after boosting were further tested for their neutralizing activities against the genetically related poxvirus VACV in both IMV and EEV forms by the 50% plaque reduction neutralization test (PRNT_50_). For IMV neutralization, IMV antigen nanoparticles M1-LuS and E8-LuS induced substantial neutralizing antibodies, with geometric mean titers (GMTs) 4155 and 884, respectively (Fig 2G). Cocktail-5 and mosaic-5 elicited substantial GMTs of 1093 and 1027, respectively (Fig 2G). In contrast, other IMV antigen nanoparticle H3-LuS and EEV antigen nanoparticles B6-LuS and A35-LuS induced poorly neutralizing antibodies against VACV IMVs (GMTs between 50–78) (Fig 2G). For EEV neutralization, B6-LuS and A35-LuS induced substantial neutralizing antibodies (GMTs 226 and 94, respectively). Notably, cocktail-5 and mosaic-5 induced significantly higher (*P* < 0.01) neutralizing antibodies compared to B6-LuS and A35-LuS (GMTs 1795 and 1119, respectively) (Fig 2H). In contrast, sera from all IMV monovalent antigen groups (M1-, E8-, and H3-LuS) poorly neutralize VACV EEV with their titers below or close to the lower detection limit.

For cellular response analysis, mice were sacrificed at 35 dpv. Their splenocytes were harvested and re-stimulated with an overlapping peptide pool of the autologous antigens for each monovalent vaccine group and the mixture of these five pools for cocktail-5, and mosaic-5. Cytokine production was examined by ELISpot assay. We found that M1-LuS and E8-LuS, cocktail-5, and mosaic-5 elicited relatively high levels of response to secret both Th1 (IFNγ and IL-2) and Th2 (IL-4) cytokines (Fig 2I). B6-LuS induced moderate cellular responses. In contrast, both H3L-LuS and A35-LuS were poorly immunogenic to elicit the production of Th1 cytokines (IFNγ and IL-2) (Fig 2I).

### Protection efficacy of MPXV nanoparticle vaccines in mice against VACV challenge

**Fig 3:**
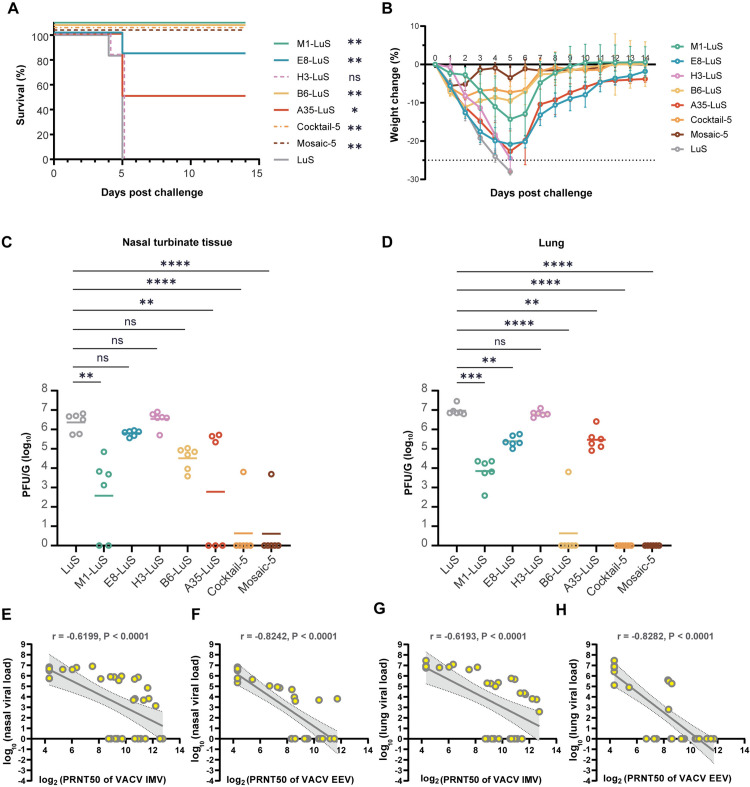
Polyvalent MPXV nanoparticle vaccines confer complete protection against VACV-WR and the neutralizing antibody titer correlates with protection. Survival curve (A) and body weight changes (B) in mice (n = 6) were monitored within 14 days after infection i.n. with 8.1 × 10^5^ CFU of VACV. Survival analysis was performed using Kaplan-Meier analysis with Log-rank test, showing significant differences between each vaccine group and sham group (ns, *P* > 0.05; *, *P* < 0.05; **, *P* < 0.01). The live virus load in the nasal turbinate tissue (C) and lung (D) of mice were examined by PRNT assay at 5 dpi. Data are shown as means ± SEM. Statistical significances were calculated by Dunn’s multiple comparison test (ns, *P* > 0.05 *, *P* < 0.05; **, *P* < 0.01; ***, *P* < 0.001; ****, *P* < 0.0001). (E-H) Correlation between neutralizing antibody titers to IMV of VACV and viral loads in nasal turbinate (E) and lung (G) tissues, between neutralizing antibody titers to EEV of VACV and viral loads in nasal turbinate (F) and lung (H) tissues. The correlations were assessed by Pearson rank-correlation tests. r and p values and a regression line of best fit are shown. Shaded area depicts the 95% CI.

Next, we evaluated the protective efficacy of these MPXV nanoparticle vaccines in mice using the well-established challenge model against the genetically related poxvirus VACV [46]. Mice were challenged with a lethal dose (30 LD_50_) of VACV (Western Reserve strain, WR) via the intranasal route at 37 dpv (Fig 2A) [46]. The body weight and survival of each mouse were recorded daily for 14 days post-infection (dpi). Tissues were collected at 5 dpi. As expected, all mice receiving the sham vaccine (LuS) succumbed (≥ 25% weight loss) to the VACV challenge between 4 and 5 dpi (Fig 3A), with severe weight loss starting from 1 dpi (Fig 3B). Consistent with the trend shown in the immunogenicity data, mice receiving the poorly immunogenic nanoparticle H3-LuS has similar decreased survival as the sham group with a slightly delayed weight loss. In contrast, mice receiving E8-LuS and A35-LuS were partially protected from death with the survival rate of 83.3% and 50%, respectively, but suffered from severe weight loss. Impressively, nanoparticles displaying IMV antigen M1 or EEV antigen B6, or multivalent particle cocktail-5 and mosaic-5 provide 100% protection from death, with less weight loss (Fig 3A and 3B).

To validate the protection efficacy against virus infection in vivo, virus burden was measured in target tissues including nasal turbinate and lung (Fig 3C and 3D). The mice with sham vaccination showed high loads of VACV in the nasal turbinate [average 3.4 × 10^6^ plaque forming units (PFU) per gram] and lung (average 1.1 × 10^7^ PFU per gram) (Fig 3C and 3D). All monovalent nanoparticles, except H3-LuS, reduced viral loads in both tissues (reduction of 0.5-3.5 order of magnitudes). Impressively, cocktail-5 and mosaic-5 showed the best protection with almost undetectable virus in both tested tissues (Fig 3C and 3D). These findings demonstrate nanoparticles presenting both IMV and EEV antigens are protective, and the multivalent antigens can achieve better efficacy compared to the monovalent ones.

The diversity in sample size and outcome variability across the different vaccine formulations enabled a comprehensive analysis of immune correlates. Pearson rank correlation was utilized to identify immune correlates of protection. The neutralizing antibody titers [log_2_(PRNT_50_)] to both IMV and EEV of VACV showed an inverse correlation with viral loads [log_10_(PFU)] in both nasal turbinates and lungs (Fig 3E-3H). In general, neutralizing antibody titers against EEV showed a stronger correlation than those against IMV in both nasal turbinates (r = −0.8242, *P* < 0.0001, and r = −0.6199, *P* < 0.0001, respectively) and lungs (r = −0.8282, *P* < 0.0001, and r = −0.6193, *P* < 0.0001, respectively).

### Immunogenicity of updated MPXV nanoparticle vaccines

**Fig 4:**
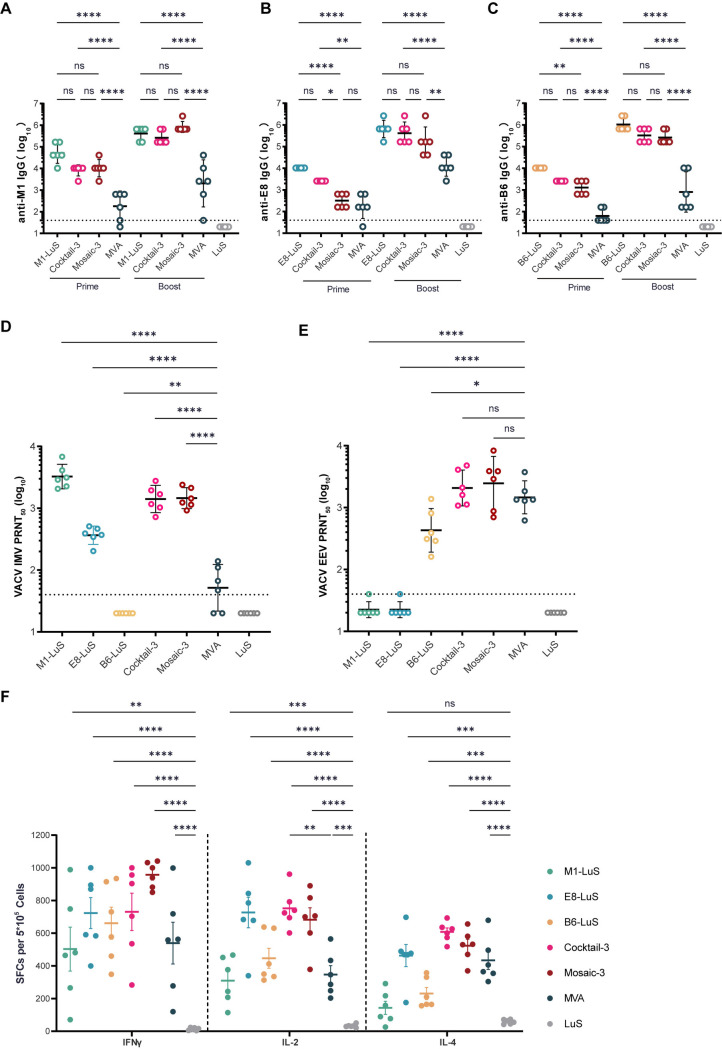
Trivalent MPXV nanoparticle vaccines induce superior immune response compared with monovalent vaccines and MVA. Mice of each group (n = 12) were immunized with monovalent vaccines (M1-LuS, E8-LuS and B6-LuS), trivalent vaccines (cocktail-3 and mosaic-3), LuS nanoparticles (2 μg/dose) or MVA (10^7^ PFU) according to the same schedule as in Fig 2A. (A-C) Antigen-specific IgG titers for MPXV antigens, including M1 (A), E8 (B), and B6 (C) were measured by ELISA in sera collected from mice (n = 6) at 19 and 35 dpv. (D-E) Neutralizing activities of sera (n = 6) against IMV (D) and EEV (E) were determined by PRNT_50_ assay against VACV infection. The data of (A-E) are GMT with a 95% CI. (F) The cellular immune response of mice (n = 6) after vaccination with two doses of each vaccine was measured by ELISpot assay with 5 × 10⁵ splenocytes per well. IFNγ, IL-2 and IL-4 secretion of splenocytes after stimulation with MPXV antigens M1, E8, and B6 overlapping peptide pools or mixture were detected to evaluate cellular immune responses. Data are group mean values ± SEM. *P* values were determined with one-way ANOVA with multiple comparison test (ns, *P* > 0.05; *, *P* < 0.05; **, *P* < 0.01; ***, *P* < 0.001; ****, *P* < 0.0001).

To minimize the antigen usage for a simpler vaccine manufacturing, the three antigens with the best immunogenicity and protection (IMV antigens M1 and E8, EEV antigen B6) were selected as the trivalent cocktail and mosaic LuS nanoparticles, namely cocktail-3 and mosaic-3, respectively, with the co-display of each antigen in the mosaic nanoparticles verified (S4 Fig). We next evaluated the immunogenicity of these new nanoparticle vaccines in BALB/c mice, according to the same immunization approach and regimen shown in Fig 2A. Two doses of 10^7^ PFUs MVA, the approved live-attenuated orthopoxviral vaccine, and monovalent M1, E8 and B6 nanoparticles were given as the controls. LuS was given as the negative control. As a result, both cocktail-3 and mosaic-3 elicited high serologic binding antibodies to those autologous antigens, with the titers comparable to those induced by the monovalent nanoparticles (Fig 4A-4C). In contrast, antibodies cross-reactive to M1, E8, and B6 elicited by MVA vaccination were significantly lower (Fig 4A-4C). We next measured the GMTs of serum neutralizing antibodies against VACV IMVs and EEVs by PRNT_50_. For IMV neutralization, the two IMV antigen-based monovalent nanoparticles (M1-LuS and E8-LuS) and trivalent nanoparticles (cocktail-3 and mosaic-3) induced high neutralizing antibodies, with the GMTs of 3255, 366, 1412, and 1459 (Fig 4D), respectively. In contrast, MVA induced significantly lower neutralizing antibodies in mice, with a GMT of 52 (Fig 4D). For EEV neutralization, the EEV antigen-based monovalent nanoparticles (B6-LuS) and trivalent nanoparticles (cocktail-3 and mosaic-3), and MVA all elicited substantial neutralizing antibodies, with the GMTs of 430, 2074, 2459, and 1465 (Fig 4E), respectively.

We also assessed the cellular immune responses in mice at 35 dpv using ELISpot assay. Splenocytes were re-stimulated with peptide pools covering autologous antigens (M1, E8, and B6) for each monovalent vaccine group and the mixture of these three pools for cocktail-3, mosaic-3, and MVA groups. The results showed that all these vaccines induced substantial secretion of both Th1 (IFNγ and IL-2) and Th2 (IL-4) cytokines from splenocytes (Fig 4F). Interestingly, cocktail-3 vaccination induced comparable levels of IFNγ, but significantly higher (*P* < 0.001) secretion of IL-2, in comparison to MVA vaccination (Fig 4F). These findings demonstrated both cocktail-3 and mosaic-3 can induce robust humoral and cellular responses to MPXV, which is comparable, if not higher, to the currently used attenuated VACV vaccine MVA.

### Protective efficacy of trivalent MPXV nanoparticle vaccines

**Fig 5:**
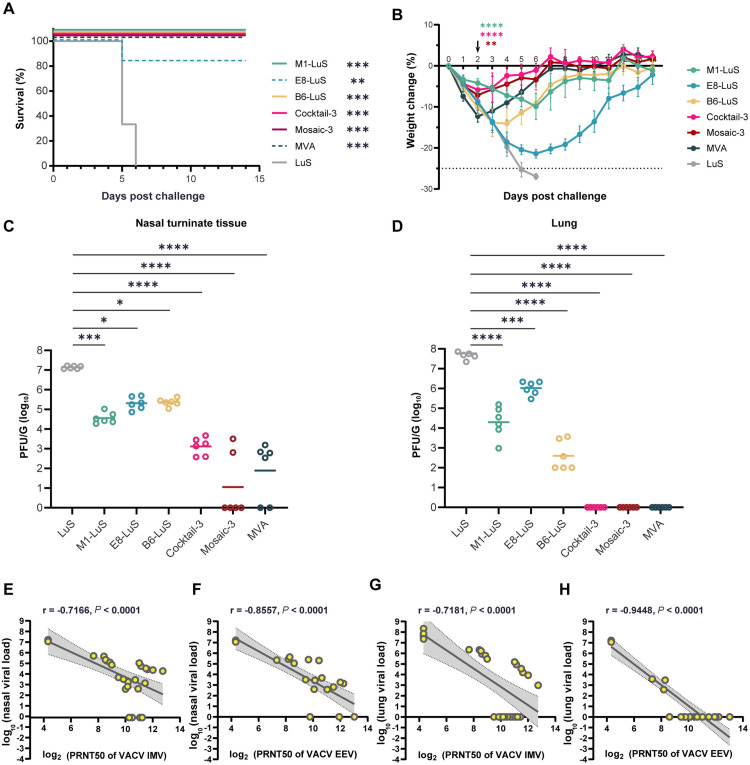
Trivalent nanoparticle vaccines and MVA confer complete protection against VACV-WR challenge. As the approach in Fig 3, the survival (A) and body weight change (B) of mice (n = 6 each group) were monitored within 14 dpi. Survival analysis was performed using Kaplan-Meier analysis with Log-rank test, showing significant differences between each vaccine group and sham group (ns, *P* > 0.05; *, *P* < 0.05; **, *P* < 0.01; ***, *P* < 0.001). *P* values of the body weight changes at 2 dpi (B) were determined with one-way ANOVA with Turkey’s multiple comparison test (ns, *P* > 0.05; *, *P* < 0.05; **, *P* < 0.01; ***, *P* < 0.001; ****, *P* < 0.0001). The tissue virus loads in the nasal turbinate (C) and lung (D) were examined by PRNT_50_ assay. Data are shown as means ± SEM. Statistical significances were calculated by one-way ANOVA with Dunn’s multiple comparison test (ns, *P* > 0.05; *, *P* < 0.05; **, *P* < 0.01; ***, *P* < 0.001; ****, *P* < 0.0001). (E-H) Correlation between neutralizing antibody titers to IMV of VACV and viral loads in nasal turbinate (E) and lung (G) tissues, between neutralizing antibody titers to EEV of VACV and viral loads in nasal turbinate (F) and lung (H) tissues of mice (n = 6). The correlations were assessed by Pearson rank-correlation tests. r and p values and a regression line of best fit are shown. Shaded area depicts the 95% CI.

The mice were challenged with a 30 LD_50_ dose of VACV-WR, and assessed for the protective efficacy of MPXV nanoparticle vaccines compared to MVA. The results showed that all mice in the LuS control group succumbed to the VACV challenge within 5 dpi (Fig 5A). In contrast, 83.3% of mice received E8-LuS survived, but with severe weight loss (Fig 5A and 5B). All mice receiving M1-LuS, B6-LuS, trivalent nanoparticles (cocktail-3 and mosaic-3), and MVA achieved 100% protection. Notably, mice in cocktail-3 and mosaic-3 groups showed significantly less weight loss compared to those receiving either monovalent nanoparticle (except M1) or MVA (Fig 5B) at 2 dpi (*P* < 0.001), suggesting the benefit of combining multivalent antigens for protection.

To validate the protection efficacy of vaccines against virus infection in vivo, we measured viral loads in respiratory tissues. As expected, all mice receiving LuS control were detected with high virus loads in the nasal turbinate (1.4 × 10^7^ PFU/g) and lung (7.6 × 10^7^ PFU/g) (Fig 5C and 5D). Compared to the LuS control group, the viral loads in the nasal turbinate were reduced by ~2 orders of magnitude in the E8-LuS (average 2.6 × 10^5^ PFU/g) and B6-LuS (average 2.5 × 10^5^ PFU/g) groups, and by ~3 orders of magnitude in the M1-LuS group (average: 4.4 × 10^4^ PFU/g) (Fig 5C). In contrast, in mice receiving cocktail-3, mosaic-3, or MVA, virus titers were reduced by ~4 orders (cocktail-3, average: 1909 PFU/g; mosaic-3, average: 638 PFU/g; MVA, average: 529 PFU/g). In regards to viral loads in the lung, vaccination with E8-LuS, M1-LuS, or B6-LuS conferred a ~ 2–4 orders of magnitude in virus reduction (average: 1.3 × 10^6^ PFU/g in E8-LuS group; 5.1 × 10^4^ PFU/g in M1-LuS group; 1.2 × 10^3^ PFU/g in B6-LuS group). Remarkably, trivalent nanoparticles (cocktail-3 and mosaic-3) and MVA conferred complete protection in the lung with undetectable viral loads (Fig 5D). These results confirmed the superior protective efficacy of the cocktail-3 and mosaic-3 nanoparticle vaccines compared to monovalent vaccines.

The Pearson rank correlation on immune protection following vaccination showed the strong inverse correlation between the neutralizing antibody titers [log_2_(PRNT_50_)] of VACV and viral loads [log_10_(PFU)] in both nasal turbinate tissue and lung (Fig 5E-5H). Again, neutralizing antibody titers to EEV showed a stronger correlation than that to IMV in both nasal turbinate (r = −0.8557, **P* *< 0.0001 and r = −0.7166, *P* < 0.0001, respectively) and lung tissues (r = −0.9448, *P* < 0.0001 and r = −0.7181, *P* < 0.0001, respectively).

### Immunogenicity and protective efficacy of a single-dose nanoparticle vaccination in female and male mice

**Fig 6:**
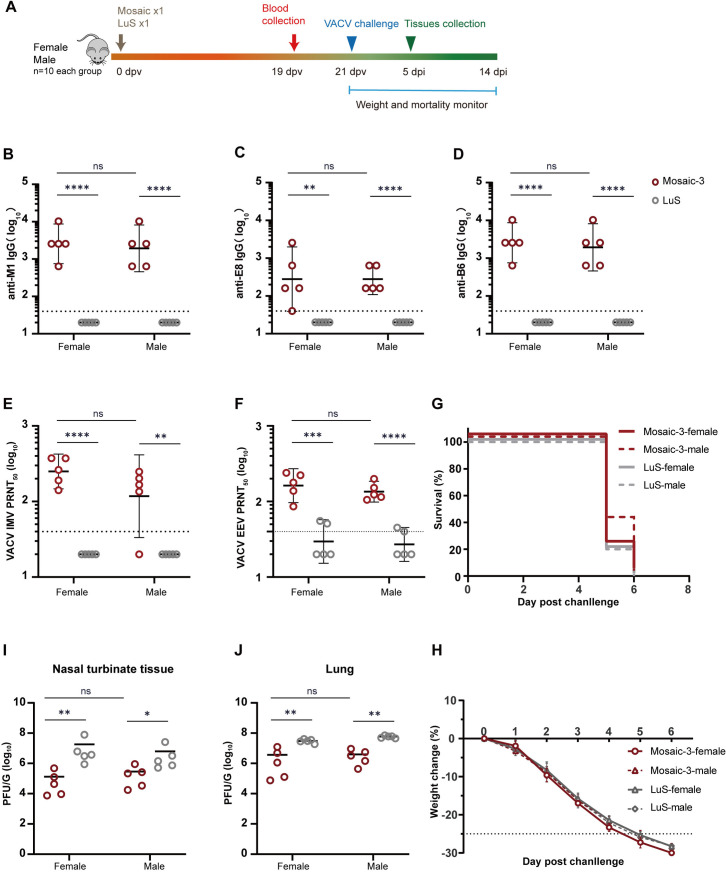
The comparison of immune responses between male and female mice and protection efficacy by single-dose vaccination. (A) Single-dose vaccination scheme for female and male mice (n = 10 each group) immunization. Mice of each group were immunized subcutaneously (s.c) with 2 μg dose of MPXV nanoparticle vaccines (mosaic-3) or with empty particles LuS and were challenged with VACV-WR at 21 dpv. Mouse icon is the seam as in Fig 2A. (B-D) Anti-specific IgG titers for MPXV antigens, including M1 (B), E8 (C), and B6 (D) were measured by ELISA in sera collected from mice (n = 5) at 19 dpv. Neutralizing activity of sera (n = 5) against IMV (E) and EEV (F) were determined by PRNT assay against VACV-WR infection, and the PRNT_50_ was calculated. The data of (B-F) are GMT with a 95% CI. *P* values of (B-F) were determined with unpaired t-test. (ns, *P* > 0.05; *, *P* < 0.05; **, *P* < 0.01; ***, *P* < 0.001; ****, *P* < 0.0001). The horizontal dashed line indicates the LLOD. The survival (G) and body weight change (H) of mice (n = 5) were monitored within 14 dpi or until losing over 25% of their initial weight. Survival analysis was performed using Kaplan-Meier analysis with Log-rank test. Mosaic-3-female vs LuS-female: *P* > 0.05 (ns), and Mosaic-3-male vs LuS-male: *P* > 0.05 (ns). The live virus load in the nasal turbinate tissue (G) and lung (H) of mice (n = 5) were examined by PRNT assay at 5 dpi. The data of (I) and (J) are shown as means ± SEM. Statistical significances of (I-J) were calculated by Mann-Whitney test (ns, *P* > 0.05; *, *P* < 0.05; **, *P* < 0.01; ***, *P* < 0.001; ****, *P* < 0.0001).

To test whether a single-dose vaccination of mpox nanoparticle is immunogenic and protective, and whether there is difference between different mice gender, we vaccinated both female and male mice a single dose of masaic-3, adjuvanted with AddaVax. LuS alone was given as sham control. Male and female mice were elicited with comparable humoral responses by 19 dpv (Fig 6A - 6D), with VACV IMV neutralization GMTs of 250 in female group and 117 in male group (p > 0.05, Fig 6E) and EEV neutralization GMTs of 162 vs 135, respectively (p > 0.05, Fig 6F). Mice were challenged intranasally with VACV-WR. Both sex groups died between 5–6 dpi with no difference of weight lost (Fig 6G and 6H) and of viral loads in nasal turbinate and lung (Fig 6I and 6J, p > 0.05). However, mosaic-3 group showed ∼1–2-log_10_ reductions of viral loads in nasal turbinate (mean reduction: 2.08, log_10_ PFU/g in females [p < 0.01] vs. 1.23 log_10_ in males [p < 0.05]) (Fig 6I) and lung (mean reduction: 1.51 log_10_ vs. 1.35 log_10_; p < 0.01) (Fig 6J) compared to LuS group. These results suggested that a single-dose of nanoparticle mpox vaccine elicited comparable antibody response between female and male mice, however, its protection is insufficient.

## Discussion

In recent years, emerging and re-emerging infectious diseases have continued to cause global health emergency [47,48]. Vaccines and monoclonal antibodies are the new cornerstones to control infectious diseases [49,50]. Since the end of 2023, a new clade Ib of the MPXV has emerged in Africa (S5 Fig), exhibiting a higher mortality rate than the MPXV clade II strain that caused an epidemic in 2022 [11]. The resurgence prompted WHO to declare a global health emergency on August 15, 2024 [10]. In response to the escalating crisis, we developed a nanoparticle-based MPXV vaccine using protective antigens from the Clade I strain (MPXV-Zeire-96). Our findings demonstrated that these nanoparticle vaccines induced robust humoral and cellular immune responses. Multivalent nanoparticles containing IMV and EEV antigens showed cross-protection against a lethal VACV challenge in mice, underscoring the potential of nanoparticles to be used in orthopoxviruses vaccine development.

The enhanced immune responses observed likely originate from several advantages of nanoparticle vaccines over traditional protein subunit vaccines. Firstly, the high-copy multivalent display of MPXV antigens on self-assembled nanoparticles LuS [42,51], ensuring dense antigen presentation [52]. Secondly, this structure and size of nanoparticles can improve B cell receptor cross-linking and persistence in the germinal center, which not only enhances the immune response of B cells but also facilitate the production of high-affinity antibodies, leading to a strong and long-lasting antibody response [37,51,53–58]. Thirdly, nanoparticles facilitate efficient uptake and processing by dendritic cells (DCs), enhancing antigen presentation and T-cell activation [40]. Resident DCs play a crucial role in T follicular helper cell (Tfh) induction and are strategically positioned to elicit rapid T cell responses to particulate antigen immunization [59]. The increase in IFNγ, IL-2, and IL-4-secreting T cells and robust immune response in mice vaccinated with MPXV multivalent nanoparticle vaccines highlights the ability of these vaccines to stimulate strong and balanced cellular immune responses (Figs 2I and 4G), essential for viral clearance.

IMV proteins M1, A29, E8, and H3, as well as EEV proteins A35 and B6, were selected as antigens based on their proven efficacy in previous studies on immunity against VARV, VACV, and MPXV [32,44,46,60–66]. Antibodies targeting these antigens have demonstrated varying levels of protection in animal studies using monoclonal and/or polyclonal antibody prophylaxis [28,32,63,67–69]. Recent research on multi-MPXV mRNA vaccines, such as a hexavalent mRNA [70], a pentavalent MPXVac-97 mRNA [45], and quadrivalent BNT166 [60] and mRNA-1769 [71], has demonstrated the immunogenicity and potential cross-protection provided by this combination of antigens. Moreover, the new development of a bivalent mRNA vaccine [35] and DAM subunit [46] has underscored the immunogenicity and protective efficacy of antigens M1 and A35. In our nanoparticle platform, M1, E8, and B6 elicited stronger specific binding and neutralizing antibodies, as well as enhanced cellular immune responses, compared to the less effective H3 and A35 antigens. VACV protein D8 (homologous to E8 of MPXV) induces strong protective antibody responses in vivo and enhances the efficacy of a multivalent poxvirus DNA vaccine [65,68]. Two neutralizing antibodies (nAbs) targeting MPXV B6 showed effective protection against VACV [72]. In accordance, trivalent nanoparticles (cocktail-3 and mosaic-3) exhibited superior protective efficacy and enhanced virus clearance in respiratory tissues compared to their autologous monovalent nanoparticles. Importantly, Importantly, the three antigens from Clade I—M1, E8, and B6—are completely conserved in the recently emerged Clade 1b (S6 Fig). This conservation suggests that cocktail-3 and mosaic-3 have the potential to effectively target the current Clade 1b strain of mpox

Notably, our study systematically analyzed the immune correlates of protection against MPXV and identified neutralizing antibodies as a key determinant for immune protection. Particularly, neutralizing antibodies against EEV and IMV showed an inverse relationship with protection, with EEV antibodies demonstrating a stronger association. These findings on antibody protection in MPXV vaccines provide valuable insights for future research on orthopoxvirus vaccine development.

In this study, we systematically compared the immune protection of nanoparticle candidate vaccines with the MVA. The trivalent nanoparticles vaccines cocktail-3 and mosaic-3 demonstrated robust protection in mice, comparable to the MVA vaccine. Interestingly, although vaccines elicited comparable neutralization antibodies of EEV and cellular immune response, cocktail-3 and mosaic-3 exhibited stronger neutralizing against IMV. Although we showed EEV-neutralizing antibodies are more closely associated with immune protection, the challenge studies revealed comparable protection between trivalent nanoparticles and MVA, and even outperformed it in terms of mitigating weight loss. Similarly, the tetravalent mRNA vaccine (mRNA-1769) also demonstrated equivalent protection to MVA-based immunity in preventing death after a lethal viral challenge, and it offered superior protection in terms of viral load reduction, weight loss mitigation, and lesion control compared to MVA-based immunity [71]. These findings highlight the potential of nanoparticle-based subunit vaccines in mitigating orthopoxvirus outbreaks.

Given men who have sex with man are the major source of mpox virus transmission, we also evaluated the vaccine immunogenicity in male animals. We showed that mosaic-3 nanoparticle elicited comparable neutralizing antibodies between male and female mice, suggesting comparable protective efficacy in between both mice genders. Besides, a single-dose vaccination is insufficient to prevent mortality induced by mpox virus infection, although substantial reduction of viral loads was observed in nasal turbinate and lung. Our results suggest a prime-boost regimen remains required to achieve full protection.

Subunit vaccines, such as those based on nanoparticles, generally have a good safety profile. A LuS-based vaccine candidates have been evaluated in clinical trials for against HIV [73] (NCT03547245). Taking this into account, cocktail-3 and mosaic-3 offer a promising alternative to the live vaccinia virus-based MVA vaccine, with safety and applicability been suitable for a broad range of individuals.

This study has several limitations. Due to the unavailability of BSL-3 facilities, we used VACV instead of live MPXV in neutralization and challenge assay of this proof-of-concept study. These approaches lay the foundation for MPXV studies in the future. Besides, the mice were challenged with virus via i.n. route. Given skin is another important avenue for transmission of both VACV and MPXV, a challenge route via skin would be required to test the vaccine efficacy in more advanced animal models, e.g., rabbit or macaques. Moreover, although mosaic manufacture is simpler compared to cocktail, ensuring the equal display of multi-antigens on the surface of nanoparticles remains challenge in quality control.

## Materials and methods

### Ethics statement

This study was carried out in accordance with the recommendations described in the Guidelines for the Care and Use of Laboratory Animals of the Institute of Microbiology, Chinese Academy of Sciences (IMCAS) Ethics Committee. All animal experiments were reviewed and approved by the Committee on the Ethics of Animal Experiments of the Institute of Microbiology, Chinese Academy of Sciences.

### Cells and viruses

Human embryonic kidney (HEK) Expi293F cells (Sino Biological) were cultured at 37°C in SMM 293-TII expression medium with 5% CO_2_. Vero cells (ATCC, CCL81), Hela cells (ATCC, CCL2.2), and BHK-21 cells (ATCC, CCL10) were cultured at 37°C in Dulbecco’s Modified Eagle medium (DMEM) (Gibco, C11995500BT) with 10% fetal bovine serum (FBS) (Gibco, 10437–028). The VACV Western Reserve (WR) was kindly provided by Prof. Min Fang from IMCAS and propagated in Vero cells. MVA (modified vaccinia virus Ankara, ATCC # VR-1508) was purchased from ATCC and amplified in BHK-21 cells.

### IMV and EEV of VACV-WR and MVA preparations

The VACV stocks were prepared according to the manufacture of the previously report [74]. All viruses were manipulated under BSL-2 conditions. VACV-WR was propagated in Vero cells using culture media (DMEM with 2% FBS, and 100 units/mL penicillin and streptomycin) as previously described [46]. After 48 hours, the cells were harvested by centrifugation at 1800 × g for 5 minutes and resuspended in culture media. The cell suspension was subjected to three complete froze-thaw to release VACV IMV particles. MVA was propagated in BHK-21 cells in DMEM with 2.5% FBS. After 72 hours, the cells were harvested and treated with the seam method allowing for release MVA particles.

As previously described [67,72], VACV EEVs were produced from HeLa cell monolayers in culture media. The cells were infected with VACV-WR at an MOI of 0.5 and the medium containing EEV was harvested after 48 hours. The supernatant was centrifuged to remove cells, then stored at 4°C and used within 2 weeks. The EEV titer was determined using an IMV-neutralizing anti-L1 antibody (7D11) [66] on Vero cells (~2 × 10^5^ PFU/mL). FBS used in all experiments was inactivated heat at 56°C for 30 min before use.

### Transient expression of SpyTag-antigens and SpyCatcher-LuS

The SpyTag-antigen constructs used for MPXV antigens include the mouse IgGκ signal peptide sequence, SpyTag peptide (16 amino acids), linker (GSG)_3_ spacer, and codon-optimized the ectodomain of *M1* (amino acid 2–183), *E8* (amino acid 2–275), *H3* (amino acid 2–282), *B6* (amino acid 20–279) and *A35* (amino acid 58–181) gene sequences, respectively, from MPXV (GenBank: AF_380138.1) fused to C-terminal hexahistidine tag (HHHHHH) into pCAGGS vector for transient expression using HEK293F cell lines. The SpyTag-antigens were produced in 800mL HEK293F cells grown in suspension using HEK293F cell complete medium (Sino Biological Inc.) at 37°C in a humidified 5% CO2 incubator rotating at 130 revolutions per minute (rpm) The supernatants were harvested, and proteins were purified from clarified supernatants using a HisTrap HP 5mL column (GE Healthcare), exchange buffer, concentrated and purified via gel filtration chromatography with HiLoad 16/600 Superdex 200 pg (GE Healthcare).

The Spy/Catcher 003-LuS expression construct consists of Spy/Catcher, linker (GSG)_3_ spacer, LuS sequence, linker (GSG)_3_, and a C-terminal hexahistidine tag (HHHHHH) into *E. Coli.* expression vector pET21a [42]. *E. Coli* strain BL21 was transformed and cultured in LB medium supplemented 100 μg/ml ampicillin for 8 hours at 37°C. For recombinant protein expression, the medium was supplemented with IPTG to a final concentration of 1 mM and shaken at 150 rpm for 3 hours at 37°C. Cells were harvested and resuspended in PBS containing 1% Triton X‐100, followed by lysis through sonication. The supernatant was collected from cell lysates by centrifugation at 16,200 g for 30 min at 4°C and through sterile syringe filters. The filter was purified by Ni affinity chromatography with a HisTrap HP 5mL column (GE Healthcare), exchange buffer, and concentrated as LuS-SC nanoparticles. The eluted nanoparticles were purified via size exclusion chromatography (SEC) with Superose 6 column (GE Healthcare) in 10 mM PBS buffer (pH 7.2). Fractions corresponding to nanoparticles were concentrated and stored at -80°C. Protein purity of these protein was assessed using SDS-PAGE.

### EM characterization of nanoparticles

Negative staining electron microscopy was employed for the characterization of nanoparticles. Specifically, 10 μl of purified nanoparticles at a concentration of 0.1 mg/mL were applied to freshly glow-discharged 300-mesh copper grids and incubated for 1 minute. The grids were then washed twice with 10 mM PBS (pH 7.2), stained with 2% (w/v) uranyl acetate, and air-dry. Imaging of the negatively stained grids was conducted using a Tecnai Spirit microscope 120 kV (120 kV TEM).

### Mouse experiments

SPF female BALB/c mice (6–8 weeks old) were procured from Beijing Vital River Animal Technology Co., Ltd and housed in an Animal Biosafety Level 2 facility at IMCAS. LuS (empty particles), M1-LuS, E8-LuS, H3-LuS, B6-LuS, A35-LuS, cocktail-5 and mosaic-5 were emulsified with AddaVax adjuvant (InvivoGen, USA) to serve as candidate vaccines. All vaccines were administered via subcutaneous (s.c) route in each group of mice with two doses of 2 μg, 21 days apart. The VACV-WR challenge was conducted at 37 dpv (30 × LD_50_, 8.1 × 10^5^ PFU) via the intranasal route (i.n.). Mouse was weighed daily for 14 dpi and euthanized after losing over 25% of their initial weight.

To evaluate humoral immunogenicity, blood samples (n = 6) were collected at 19 and 35 dpv. Serum was separated from blood samples by centrifugation and stored at -80 °C until testing.

To evaluate cellular immunogenicity, spleens (n = 6) were collected at 35 dpv after sacrificing mice. The spleens were homogenized using a tissue grinder and filtered through a 40 μm cell strainer (Corning). Red blood cells were lysed with a red blood cell lysis buffer (Solarbio Life Science). Splenocytes were stained with acridine orange/propidium iodide (AO/PI) and counted using a cell counter (Count star). Live splenocytes were then immediately used for the ELISpot assay.

For the collection of nasal turbinate tissue and lung tissues, the mice (n = 6) were euthanized and necropsied at 5 dpi. Tissue samples were prepared for virus titer determination. The tissues were weighed, placed in 500 μl of serum-free RPMI 1640, homogenized and centrifugated at 212 g for 10 minutes. The supernatants were serially diluted tenfold (started at 1:10) and inoculated onto Vero cells in 12-well plates [46]. After 1 hour of incubation the medium was removed, the wells were washed once with PBS, and then overlaid with culture media containing 1% carboxymethylcellulose. The plates were incubated for 2 days and then plaques were visualized by crystal violet staining. Viral load was calculated based on plaque numbers and expressed as PFU per gram of tissue. If no infection was observed, an arbitrary titer value (100 PFU/g) of the lower limit of detection was reported based on the specific assay conditions.

To determine whether vaccine efficacy was influenced by sex, an additional cohort of male BALB/c mice (7 weeks old, n = 10) was vaccinated in parallel with females under identical conditions (single 2 µg dose). Sera were collected on 19 dpv for antigen-specific IgG ELISA and VACV neutralization tests. On 21 dpv, mice were challenged i.n. with 30 × LD₅₀ VACV-WR; nasal turbinates and lungs were harvested at 5 dpi for PFU quantification as above, and weighted daily within14 dpi or until losing over 25% of their initial weight.

### Enzyme-linked immunosorbent assay (ELISA)

The binding properties of sera to each antigen were determined by ELISA as previously described [60], with some modifications. Briefly, 96-well plates were coated over-night with 3 μg/ml of M1, E8, H3, B6, and A35 protein, respectively, in 0.05 M carbonate-bicarbonate buffer (pH 9.6) and blocked in 5% skim milk in PBS. Serum samples from mice were serially diluted and added to each well. The plates were incubated for 2 hours and then washed. The plates were incubated with goat anti-mouse IgG-HRP antibody for 1.5 hours and then washed. The plates subsequently developed with 3,3’,5,5’-tetramethylbenzidine (TMB) substrate. Reactions were stopped with 2 M hydrochloric acid, and the absorbance was measured at 450 nm using a microplate reader (PerkinElmer, USA). The endpoint titers were defined as the highest reciprocal dilution of serum to give an absorbance greater than 2.5-fold of the background values. Antibody titer below the limit of detection was determined as half the limit of detection.

### Plaque reduction neutralization test (PRNT) assay

For serum neutralization, prepare IMVs contained in DMEM (4% FBS) or EEVs containing an additional 100 μg/ml of mAb 7D11 and 20% rabbit complement (Cedariane, Canada) with ~150 PFU, as described in previous studies [46,72]. Serum samples were serially diluted two-fold starting at 1:20 using DMEM and mixed with an equal volume of IMVs or EEVs and incubated for 2 h at 37°C. The mixture was transferred into a 12-well plate containing a confluent monolayer of Vero cells. After 1 hour of incubation at 37°C in a 5% CO_2_ atmosphere. The cells were washed once with PBS, overlaid with culture media containing 1% Carboxymethylcellulose and 100 U/ml penicillin and 100 μg/ml streptomycin. The plates were incubated for 48 hours and then fixed with 4% Paraformaldehyde (Solarbio Life science) for 2 hours, and stained with 0.5% crystal violet overnight. Plaques were captured and calculated by ELISpot reader and BioSpot image analysis software. The PRNT_50_ was calculated using Prism (GraphPad) through the log(inhibitor) versus normalized response with variable slope.

### ELISpot assays

Following the manufacturer’s instructions, ELISpot assays based on IFNγ, IL-2, and IL-4 were performed using mouse IFNγ, IL-2, and IL-4 ELISpot kits (Mabtech). Briefly, spleens from mice were collected at 35 days after the prime vaccination. MultiScreen HTS IP Filter Plates (Millipore Sigma #MSIPS4W10) were precoated overnight at 4°C with anti-mouse IFNγ, IL-2, or IL-4 antibody. The plates were then washed twice and blocked for 1 hour with RPMI 1640 medium at room temperature. 1–5 × 10⁵ cells/well splenocytes were plated and re-stimulated with each peptide pool (2 μg/ml for each peptide). After a 40 hours incubation at 37°C, the cells were removed, and the plates were sequentially processed with biotinylated detection antibody, streptavidin-ALP conjugate, and substrate. The number of spots was quantified using an automatic ELISpot reader and image analysis software (Immuno Capture 6.5.0). In the IFNγ ELISpot assay, the high density of spots in some wells exceeded the detection limit of the automated reader, leading to potential inaccuracies in spot enumeration. To ensure consistent and reliable quantification, a maximum spot count of 1000 was custom-defined for wells with spot densities beyond the machine’s recognition capability.

### Biolayer interferometry assay (BLI)

The protein binding kinetics assay was performed using on an Octet RED 96 biosensor using either an anti-mouse IgG Fc capture (AMC) or a ProA biosensor. The AMC biosensor was coated with the VACV L1 (corresponding MPXV homologous antigen M1) neutralizing mAb 7D11, and ProA biosensors were coated with E8 specific mAb 8A11, H3 specific mAb 13A3, B6 specific mAb 22F, or A35 specific mAb 10H6. Except 7D11, other mAbs were kindly provided by Dr. Li Yan from IMCAS. Antibodies were diluted in PBST buffer to a final concentration of 5 µg/mL, captured on the biosensor’s surface within 180 seconds, and reached baseline levels. The biosensor underwent an association step with 200 nM MPXV antigen for 900 seconds, followed by a dissociation phase lasting for 200 seconds. Raw data were processed to eliminate baseline interference using Octet Data Analysis 7.0 software from Pall Forte Bio and then exported to GraphPad Prism 9.0 for curve fitting of the association using global linear regression analysis.

### Statistical analysis

For the ELISA and PRNT assays, data were presented as geometric means with 95% confidence interval (CI). The statistics for the ELISpot assay, weight changes, and virus loads of respiratory tissues were presented as mean ± standard error of the mean. For the survival curve, statistical analysis was performed using Kaplan-Meier with the log-rank test. Other statistical analyses were performed using one-way ANOVA with multiple-comparison tests or t-test. Correlation analysis was performed using Pearson rank correlation tests. P < 0.05 was considered statistically significant. All graphs and statistical analyses were generated using GraphPad version 9.0 software. The applied analytical methods and statistical significance are indicated in the corresponding legends.

## Supporting information

S1 Fig Homology alignment analysis of the antigenic sequences of MPXV and VACV. **(A-E)** Shown are sequence alignments of mpox clade I virus (MPXV-Zaire-96) strain proteins against orthologous proteins from vaccinia virus strain WR (VACV-WR) wild type for M1 to L1 **(A)**, E8 to D8 **(B)**, H3 to H3 **(C)**, B6 to B5 **(D)**, and A35 to A33 **(E)**. And Amino acid identity is 98.4% for M1/L1, 94.07% for E8/D8, 93.83% for H3/H3, 96.53% for B6/B5, and 93.5% for A33/A35.
(TIF)

S2 Fig Characterization and confirmation of MPXV nanoparticle vaccines by analytical gel filtration and gel electrophoresis. Size-exclusion chromatography trace for LuS shown via dotted black line, M1-LuS **(A)**, E8-LuS **(B)**, H3-LuS **(C)**, B6-LuS **(D)**, A35-LuS **(E)** and mosaic-5 **(F)** shown via solid color lines, and single antigen purified by HiLoad 16/600 Superdex 200 pg shown via corresponding color dotted line as control. Each antigen-LuS nanoparticle exhibited peak forward shifts of retention. The reducing SDS-PAGE analysis for the purified antigens, LuS, M1-LuS **(A)**, E8-LuS **(B)**, H3-LuS **(C)**, B6-LuS **(D)**, A35-LuS **(E)** nanoparticles, and each individual antigen.
(TIF)

S3 Fig Confirmation of MPXV antigens presented on mosaic-5 nanoparticles using BLI. Nanoparticle immunogens and each antigen were associated with VACV L1-specific neutralizing mAb 7D11 for corresponding MPXV homologous antigen M1 **(A)** captured on AMC biosensors, and with E8 specific mAb 8A11 **(B)**, H3 specific mAb 13A3 **(C)**, B6 specific mAb 22F9 **(D)**, and A35 specific mAb 10H6 **(E)** captured on ProA biosensors for 900 s, respectively.
(TIF)

S4 Fig Characteristics of the MPXV mosaic-3 nanoparticle. Analytical gel filtration profiles **(A)**, SDS-PAGE analysis **(B)**, and Negative-staining Ems **(C)** of mosaic were shown. Size-exclusion chromatography trace for LuS shown via dotted black line, mosaic-3 shown via solid color lines. And mosaic-3 nanoparticle exhibited peak forward shifts of retention.
(TIF)

S5 Fig Phylogenetic tree of Monkeypox virus (MPXV) isolates from 2022 to 2024. The phylogenetic tree illustrates the evolutionary relationships among various MPXV isolates collected between 2022 and 2024. The tree including 118 sequences from different geographical locations, primarily Nigeria, South Africa, Cameroon, Egypt, Sudan, and the Democratic Republic of the Congo downloaded and analysis by National Center for Biotechnology Information (NCBI). The isolates are grouped into two main clades: Clade II (Black), corresponding to the period from May 2022 to May 2023, and Clade Ib (Red), representing the interval from October 2023 to January 2024. This classification reflects distinct evolutionary lineages.
(TIF)

S6 Fig Conservative analysis of M1, E8, and B6 between MPXV Clade I and Clade 1b strains. **(A-C)** Shown are sequence alignments between Clade I strain (MPXV-Zaire-96, accession AF380138) and Clade Ib strain (MPXV-DRC-2024, accession PP601224) for M1 **(A)**, E8 **(B)** and B6 **(C)**. The amino acid identities of three proteins are completely conserved.
(TIF)

## References

[1] Luna N, Ramírez AL, Muñoz M, Ballesteros N, Patiño LH, Castañeda SA, et al. Phylogenomic analysis of the monkeypox virus (MPXV) 2022 outbreak: Emergence of a novel viral lineage? Travel Med Infect Dis. 2022;49:102402. doi: 10.1016/j.tmaid.2022.102402 35840078 PMC9628808

[2] Yang S, Xia C, Zhang Y, Shen Y, Xia C, Lu Y, et al. Clinical features and viral load variations of Mpox: a retrospective study in Chongqing, China. BMC Infect Dis. 2024;24(1):641. doi: 10.1186/s12879-024-09537-0 38926635 PMC11202379

[3] Gessain A, Nakoune E, Yazdanpanah Y. Monkeypox. N Engl J Med. 2022;387(19):1783–93. 36286263 10.1056/NEJMra2208860

[4] Thornhill JP, Barkati S, Walmsley S, Rockstroh J, Antinori A, Harrison LB, et al. Monkeypox Virus Infection in Humans across 16 Countries - April-June 2022. N Engl J Med. 2022;387(8):679–91. doi: 10.1056/NEJMoa2207323 35866746

[5] WHO. Multi-country outbreak of mpox, External situation report#32- 30 April 2024. 2024. Available from: https://wwwwhoint/.

[6] Nuzzo JB, Borio LL, Gostin LO. The WHO Declaration of Monkeypox as a Global Public Health Emergency. JAMA. 2022;328(7):615–7. doi: 10.1001/jama.2022.12513 35895041

[7] Ghebreyesus TA. Fifth Meeting of the International Health Regulations (2005) (IHR) Emergency Committee on the Multi-Country Outbreak of Mpox (Monkeypox). 2023.

[8] WHO. Mpox (Monkeypox) in the Democratic Republic of the Congo. 2023.

[9] WHO. Multi-country outbreak of mpox, External situation report #35. 2024; Available from: https://www.who.int/publications/m/item/multi-country-outbreak-of-mpox--external-situation-report-35--12-august-2024

[10] Kozlov M. Growing mpox outbreak prompts WHO to declare global health emergency. Nature. 2024. 10.1038/d41586-024-02607-y 39143282

[11] Vakaniaki EH, Kacita C, Kinganda-Lusamaki E, O’Toole A, Wawina-Bokalanga T, Mukadi-Bamuleka D. Sustained human outbreak of a new MPXV clade I lineage in eastern Democratic Republic of the Congo. Nat Med. 2024. 10.1038/s41591-024-03130-3 PMC11485229 38871006

[12] WHO. 2022-24 Mpox (Monkeypox) Outbreak: Global Trends. 2025. Available from: https://worldhealthorg.shinyapps.io/mpx_global/#1_Overview

[13] Shchelkunov SN, Totmenin AV, Safronov PF, Mikheev MV, Gutorov VV, Ryazankina OI, et al. Analysis of the monkeypox virus genome. Virology. 2002;297(2):172–94. doi: 10.1006/viro.2002.1446 12083817 PMC9534300

[14] Kugelman JR, Johnston SC, Mulembakani PM, Kisalu N, Lee MS, Koroleva G, et al. Genomic variability of monkeypox virus among humans, Democratic Republic of the Congo. Emerg Infect Dis. 2014;20(2):232–9. doi: 10.3201/eid2002.130118 24457084 PMC3901482

[15] Fenner FHD, Arita I, Jezek Z, Ladnyi ID. Smallpox and its eradication. Geneva: World Health Organization. 1988.

[16] FDA. Mpox. 2024. Available from: https://wwwfdagov/emergency-preparedness-and-response/mcm-issues/mpox

[17] Overton ET, Lawrence SJ, Wagner E, Nopora K, Rösch S, Young P, et al. Immunogenicity and safety of three consecutive production lots of the non replicating smallpox vaccine MVA: A randomised, double blind, placebo controlled phase III trial. PLoS One. 2018;13(4):e0195897. doi: 10.1371/journal.pone.0195897 29652929 PMC5898760

[18] Turner Overton E, Schmidt D, Vidojkovic S, Menius E, Nopora K, Maclennan J, et al. A randomized phase 3 trial to assess the immunogenicity and safety of 3 consecutively produced lots of freeze-dried MVA-BN® vaccine in healthy adults. Vaccine. 2023;41(2):397–406. doi: 10.1016/j.vaccine.2022.10.056 36460535 PMC9707699

[19] Prevention CFDCa. Patient’s guide to mpox treatment with tecovirimat (TPOXX). 2023; [cited October 23, 2023]. Available from: https://www.cdc.gov/poxvirus/mpox/if-sick/treatment.html

[20] Zaeck LM, Lamers MM, Verstrepen BE, Bestebroer TM, van Royen ME, Götz H, et al. Low levels of monkeypox virus-neutralizing antibodies after MVA-BN vaccination in healthy individuals. Nat Med. 2023;29(1):270–8. doi: 10.1038/s41591-022-02090-w 36257333 PMC9873555

[21] Deputy NP, Deckert J, Chard AN, Sandberg N, Moulia DL, Barkley E, et al. Vaccine Effectiveness of JYNNEOS against Mpox Disease in the United States. N Engl J Med. 2023;388(26):2434–43. doi: 10.1056/NEJMoa2215201 37199451 PMC10962869

[22] Tomita N, Terada-Hirashima J, Uemura Y, Shimizu Y, Iwasaki H, Yano R, et al. An open-label, non-randomized study investigating the safety and efficacy of smallpox vaccine, LC16, as post-exposure prophylaxis for mpox. Hum Vaccin Immunother. 2023;19(2):2242219. doi: 10.1080/21645515.2023.2242219 37559375 PMC10416734

[23] Sah R, Paul D, Mohanty A, Shah A, Mohanasundaram AS, Padhi BK. Monkeypox (Mpox) vaccines and their side effects: the other side of the coin. Int J Surg. 2023;109(2):215–7. doi: 10.1097/JS9.0000000000000142 36799858 PMC10389550

[24] Pischel L, Martini BA, Yu N, Cacesse D, Tracy M, Kharbanda K, et al. Vaccine effectiveness of 3rd generation mpox vaccines against mpox and disease severity: A systematic review and meta-analysis. Vaccine. 2024;42(25):126053. doi: 10.1016/j.vaccine.2024.06.021 38906763

[25] Bengali Z, Satheshkumar PS, Moss B. Orthopoxvirus species and strain differences in cell entry. Virology. 2012;433(2):506–12. doi: 10.1016/j.virol.2012.08.044 22999097 PMC3470877

[26] Li H, Huang Q-Z, Zhang H, Liu Z-X, Chen X-H, Ye L-L, et al. The land-scape of immune response to monkeypox virus. EBioMedicine. 2023;87:104424. doi: 10.1016/j.ebiom.2022.104424 36584594 PMC9797195

[27] Moss B. Smallpox vaccines: targets of protective immunity. Immunol Rev. 2011;239(1):8–26. doi: 10.1111/j.1600-065X.2010.00975.x 21198662 PMC3074351

[28] Golden JW, Zaitseva M, Kapnick S, Fisher RW, Mikolajczyk MG, Ballantyne J, et al. Polyclonal antibody cocktails generated using DNA vaccine technology protect in murine models of orthopoxvirus disease. Virol J. 2011;8:441. doi: 10.1186/1743-422X-8-441 21933385 PMC3192780

[29] Hatmal MM, Al-Hatamleh MAI, Olaimat AN, Ahmad S, Hasan H, Ahmad Suhaimi NA, et al. Comprehensive literature review of monkeypox. Emerg Microbes Infect. 2022;11(1):2600–31. doi: 10.1080/22221751.2022.2132882 36263798 PMC9627636

[30] Sagdat K, Batyrkhan A, Kanayeva D. Exploring monkeypox virus proteins and rapid detection techniques. Front Cell Infect Microbiol. 2024;14:1414224. doi: 10.3389/fcimb.2024.1414224 38863833 PMC11165096

[31] Hooper JW, Custer DM, Thompson E. Four-gene-combination DNA vaccine protects mice against a lethal vaccinia virus challenge and elicits appropriate antibody responses in nonhuman primates. Virology. 2003;306(1):181–95. doi: 10.1016/s0042-6822(02)00038-7 12620810 PMC9628742

[32] Gilchuk I, Gilchuk P, Sapparapu G, Lampley R, Singh V, Kose N, et al. Cross-Neutralizing and Protective Human Antibody Specificities to Poxvirus Infections. Cell. 2016;167(3):684-694.e9. doi: 10.1016/j.cell.2016.09.049 27768891 PMC5093772

[33] Hooper JW, Thompson E, Wilhelmsen C, Zimmerman M, Ichou MA, Steffen SE, et al. Smallpox DNA vaccine protects nonhuman primates against lethal monkeypox. J Virol. 2004;78(9):4433–43. doi: 10.1128/jvi.78.9.4433-4443.2004 15078924 PMC387704

[34] Mucker EM, Golden JW, Hammerbeck CD, Kishimori JM, Royals M, Joselyn MD, et al. A Nucleic Acid-Based Orthopoxvirus Vaccine Targeting the Vaccinia Virus L1, A27, B5, and A33 Proteins Protects Rabbits against Lethal Rabbitpox Virus Aerosol Challenge. J Virol. 2022;96(3):e0150421. doi: 10.1128/JVI.01504-21 34851148 PMC8826804

[35] Hou F, Zhang Y, Liu X, Murad YM, Xu J, Yu Z, et al. mRNA vaccines encoding fusion proteins of monkeypox virus antigens protect mice from vaccinia virus challenge. Nat Commun. 2023;14(1):5925. doi: 10.1038/s41467-023-41628-5 37739969 PMC10516993

[36] Tamir H, Noy-Porat T, Melamed S, Cherry-Mimran L, Barlev-Gross M, Alcalay R, et al. Synergistic effect of two human-like monoclonal antibodies confers protection against orthopoxvirus infection. Nat Commun. 2024;15(1):3265. doi: 10.1038/s41467-024-47328-y 38627363 PMC11021552

[37] Al-Halifa S, Gauthier L, Arpin D, Bourgault S, Archambault D. Nanoparticle-Based Vaccines Against Respiratory Viruses. Front Immunol. 2019;10:22. doi: 10.3389/fimmu.2019.00022 30733717 PMC6353795

[38] Joyce MG, King HAD, Elakhal-Naouar I, Ahmed A, Peachman KK, Macedo Cincotta C, et al. A SARS-CoV-2 ferritin nanoparticle vaccine elicits protective immune responses in nonhuman primates. Sci Transl Med. 2022;14(632):eabi5735. doi: 10.1126/scitranslmed.abi5735 34914540

[39] Boyoglu-Barnum S, Ellis D, Gillespie RA, Hutchinson GB, Park Y-J, Moin SM, et al. Quadrivalent influenza nanoparticle vaccines induce broad protection. Nature. 2021;592(7855):623–8. doi: 10.1038/s41586-021-03365-x 33762730 PMC8269962

[40] Wang W, Zhou X, Bian Y, Wang S, Chai Q, Guo Z, et al. Dual-targeting nanoparticle vaccine elicits a therapeutic antibody response against chronic hepatitis B. Nat Nanotechnol. 2020;15(5):406–16. doi: 10.1038/s41565-020-0648-y 32123380 PMC7223715

[41] Cohen AA, van Doremalen N, Greaney AJ, Andersen H, Sharma A, Starr TN, et al. Mosaic RBD nanoparticles protect against challenge by diverse sarbecoviruses in animal models. Science. 2022;377(6606):eabq0839. doi: 10.1126/science.abq0839 35857620 PMC9273039

[42] Liu C, Xu S, Zheng Y, Xie Y, Xu K, Chai Y, et al. Mosaic RBD nanoparticle elicits immunodominant antibody responses across sarbecoviruses. Cell Rep. 2024;43(5):114235. doi: 10.1016/j.celrep.2024.114235 38748880

[43] Zakeri B, Fierer JO, Celik E, Chittock EC, Schwarz-Linek U, Moy VT, et al. Peptide tag forming a rapid covalent bond to a protein, through engineering a bacterial adhesin. Proc Natl Acad Sci U S A. 2012;109(12):E690-7. doi: 10.1073/pnas.1115485109 22366317 PMC3311370

[44] Gao F, He C, Liu M, Yuan P, Tian S, Zheng M. Cross-reactive immune responses to monkeypox virus induced by MVA vaccination in mice. Virol J. 2023;20(1):126. 37337226 10.1186/s12985-023-02085-0 PMC10278293

[45] Fang Z, Monteiro VS, Renauer PA, Shang X, Suzuki K, Ling X, et al. Polyvalent mRNA vaccination elicited potent immune response to monkeypox virus surface antigens. Cell Res. 2023;33(5):407–10. doi: 10.1038/s41422-023-00792-5 36879038 PMC9988199

[46] Wang H, Yin P, Zheng T, Qin L, Li S, Han P, et al. Rational design of a “two-in-one” immunogen DAM drives potent immune response against mpox virus. Nat Immunol. 2024;25(2):307–15. doi: 10.1038/s41590-023-01715-7 38182667

[47] Liu J, Gao GF, Yuen K-Y, Leo Poon LM, Song N. Time is now: Preparing for the next pandemic. hLife. 2025;3(3):113–7. doi: 10.1016/j.hlife.2024.12.008

[48] Gao GF, Hoffmann JA, Walzer C, Lu J. Global public health crisis response: A roundtable discussion with Professor George Fu Gao, Professor Jules A Hoffmann, Professor Chris Walzer and Professor Jiahai Lu. hLife. 2023;1(2):63–70. doi: 10.1016/j.hlife.2023.10.001

[49] Dai L, Gao GF. Viral targets for vaccines against COVID-19. Nat Rev Immunol. 2021;21(2):73–82. doi: 10.1038/s41577-020-00480-0 33340022 PMC7747004

[50] Dai L, Song J, Xu L, Gao Z, Xu S, Chai Y, et al. A protective human antibody against respiratory syncytial virus by targeting a prefusion epitope across sites IV and V of the viral fusion glycoprotein. hLife. 2023;1(1):12–25. doi: 10.1016/j.hlife.2023.09.003

[51] Cohen AA, Gnanapragasam PNP, Lee YE, Hoffman PR, Ou S, Kakutani LM, et al. Mosaic nanoparticles elicit cross-reactive immune responses to zoonotic coronaviruses in mice. Science. 2021;371(6530):735–41. doi: 10.1126/science.abf6840 33436524 PMC7928838

[52] Yan H, Peng Y, Zhang J, Peng R, Feng X, Su J, et al. Rapid and highly potent humoral responses to mpox nanovaccine candidates adjuvanted by thermostable scaffolds. Vaccine. 2024;42(8):2072–80. doi: 10.1016/j.vaccine.2024.02.027 38423815

[53] Badten AJ, Ramirez A, Hernandez-Davies JE, Albin TJ, Jain A, Nakajima R, et al. Protein Nanoparticle-Mediated Delivery of Recombinant Influenza Hemagglutinin Enhances Immunogenicity and Breadth of the Antibody Response. ACS Infect Dis. 2023;9(2):239–52. doi: 10.1021/acsinfecdis.2c00362 36607269 PMC9926493

[54] Abbott RK, Lee JH, Menis S, Skog P, Rossi M, Ota T, et al. Precursor Frequency and Affinity Determine B Cell Competitive Fitness in Germinal Centers, Tested with Germline-Targeting HIV Vaccine Immunogens. Immunity. 2018;48(1):133-146.e6. doi: 10.1016/j.immuni.2017.11.023 29287996 PMC5773359

[55] Bachmann MF, Jennings GT. Vaccine delivery: a matter of size, geometry, kinetics and molecular patterns. Nat Rev Immunol. 2010;10(11):787–96. doi: 10.1038/nri2868 20948547

[56] Marcandalli J, Fiala B, Ols S, Perotti M, de van der Schueren W, Snijder J, et al. Induction of Potent Neutralizing Antibody Responses by a Designed Protein Nanoparticle Vaccine for Respiratory Syncytial Virus. Cell. 2019;176(6):1420-1431.e17. doi: 10.1016/j.cell.2019.01.046 30849373 PMC6424820

[57] Kasturi SP, Skountzou I, Albrecht RA, Koutsonanos D, Hua T, Nakaya HI, et al. Programming the magnitude and persistence of antibody responses with innate immunity. Nature. 2011;470(7335):543–7. doi: 10.1038/nature09737 21350488 PMC3057367

[58] Tam HH, Melo MB, Kang M, Pelet JM, Ruda VM, Foley MH, et al. Sustained antigen availability during germinal center initiation enhances antibody responses to vaccination. Proc Natl Acad Sci U S A. 2016;113(43):E6639–48. doi: 10.1073/pnas.1606050113 27702895 PMC5086995

[59] Eisenbarth SC. Dendritic cell subsets in T cell programming: location dictates function. Nat Rev Immunol. 2019;19(2):89–103. doi: 10.1038/s41577-018-0088-1 30464294 PMC7755085

[60] Zuiani A, Dulberger CL, De Silva NS, Marquette M, Lu Y-J, Palowitch GM, et al. A multivalent mRNA monkeypox virus vaccine (BNT166) protects mice and macaques from orthopoxvirus disease. Cell. 2024;187(6):1363-1373.e12. doi: 10.1016/j.cell.2024.01.017 38366591

[61] Otter AD, Jones S, Hicks B, Bailey D, Callaby H, Houlihan C. Monkeypox virus-infected individuals mount comparable humoral immune responses as smallpox-vaccinated individuals. Nature Communications. 2023;14(1):5948. 10.1038/s41467-023-41587-x PMC10517934 37741831

[62] Ye T, Zhou J, Guo C, Zhang K, Wang Y, Liu Y, et al. Polyvalent mpox mRNA vaccines elicit robust immune responses and confer potent protection against vaccinia virus. Cell Rep. 2024;43(6):114269. doi: 10.1016/j.celrep.2024.114269 38787725

[63] Fantin RF, Coelho CH. Human antibody responses to circulating monkeypox virus emphasise the need for the first mpox-specific vaccine. Lancet Microbe. 2024;5(3):e204–5. doi: 10.1016/S2666-5247(23)00365-8 38219760

[64] Freyn AW, Atyeo C, Earl PL, Americo JL, Chuang GY, Natarajan H, et al. An mpox virus mRNA-lipid nanoparticle vaccine confers protection against lethal orthopoxviral challenge. Sci Transl Med. 2023;15(716):eadg3540. 10.1126/scitranslmed.adg3540 37792954

[65] Berhanu A, Wilson RL, Kirkwood-Watts DL, King DS, Warren TK, Lund SA, et al. Vaccination of BALB/c mice with Escherichia coli-expressed vaccinia virus proteins A27L, B5R, and D8L protects mice from lethal vaccinia virus challenge. J Virol. 2008;82(7):3517–29. doi: 10.1128/JVI.01854-07 18199639 PMC2268497

[66] Wolffe EJ, Vijaya S, Moss B. A myristylated membrane protein encoded by the vaccinia virus L1R open reading frame is the target of potent neutralizing monoclonal antibodies. Virology. 1995;211(1):53–63. doi: 10.1006/viro.1995.1378 7645236

[67] Benhnia MR-E-I, McCausland MM, Moyron J, Laudenslager J, Granger S, Rickert S, et al. Vaccinia virus extracellular enveloped virion neutralization in vitro and protection in vivo depend on complement. J Virol. 2009;83(3):1201–15. doi: 10.1128/JVI.01797-08 19019965 PMC2620895

[68] Sakhatskyy P, Wang S, Chou T-HW, Lu S. Immunogenicity and protection efficacy of monovalent and polyvalent poxvirus vaccines that include the D8 antigen. Virology. 2006;355(2):164–74. doi: 10.1016/j.virol.2006.07.017 16919703 PMC7126721

[69] Lustig S, Fogg C, Whitbeck JC, Eisenberg RJ, Cohen GH, Moss B. Combinations of polyclonal or monoclonal antibodies to proteins of the outer membranes of the two infectious forms of vaccinia virus protect mice against a lethal respiratory challenge. J Virol. 2005;79(21):13454–62. doi: 10.1128/JVI.79.21.13454-13462.2005 16227266 PMC1262616

[70] Zhang N, Cheng X, Zhu Y, Mo O, Yu H, Zhu L, et al. Multi-valent mRNA vaccines against monkeypox enveloped or mature viron surface antigens demonstrate robust immune response and neutralizing activity. Sci China Life Sci. 2023;66(10):2329–41. doi: 10.1007/s11427-023-2378-x 37300753 PMC10257374

[71] Mucker EM, Freyn AW, Bixler SL, Cizmeci D, Atyeo C, Earl PL, et al. Comparison of protection against mpox following mRNA or modified vaccinia Ankara vaccination in nonhuman primates. Cell. 2024;187(20):5540–53.e10. doi: 10.1016/j.cell.2024.08.043 39236707

[72] Zhao R, Wu L, Sun J, Liu D, Han P, Gao Y, et al. Two noncompeting human neutralizing antibodies targeting MPXV B6 show protective effects against orthopoxvirus infections. Nat Commun. 2024;15(1):4660. doi: 10.1038/s41467-024-48312-2 38821921 PMC11143242

[73] Leggat DJ, Cohen KW, Willis JR, Fulp WJ, deCamp AC, Kalyuzhniy O. Vaccination induces HIV broadly neutralizing antibody precursors in humans. Science. 2022;378(6623):eadd6502. 10.1126/science.add6502 PMC11103259 36454825

[74] Cotter CA, Earl PL, Wyatt LS, Moss B. Preparation of Cell Cultures and Vaccinia Virus Stocks. Curr Protoc Protein Sci. 2017;89:5.12.1–5.12.18. doi: 10.1002/cpps.34 28762495

