# Supplementary figures and images for: Multivalent mpox protein nanoparticle vaccines confer cross-protection against orthopoxvirus infection
Source: PLoS Pathog. 2025 Aug 21;21(8):e1013389. doi: 10.1371/journal.ppat.1013389 (PMC12370032; doi:10.1371/journal.ppat.1013389)

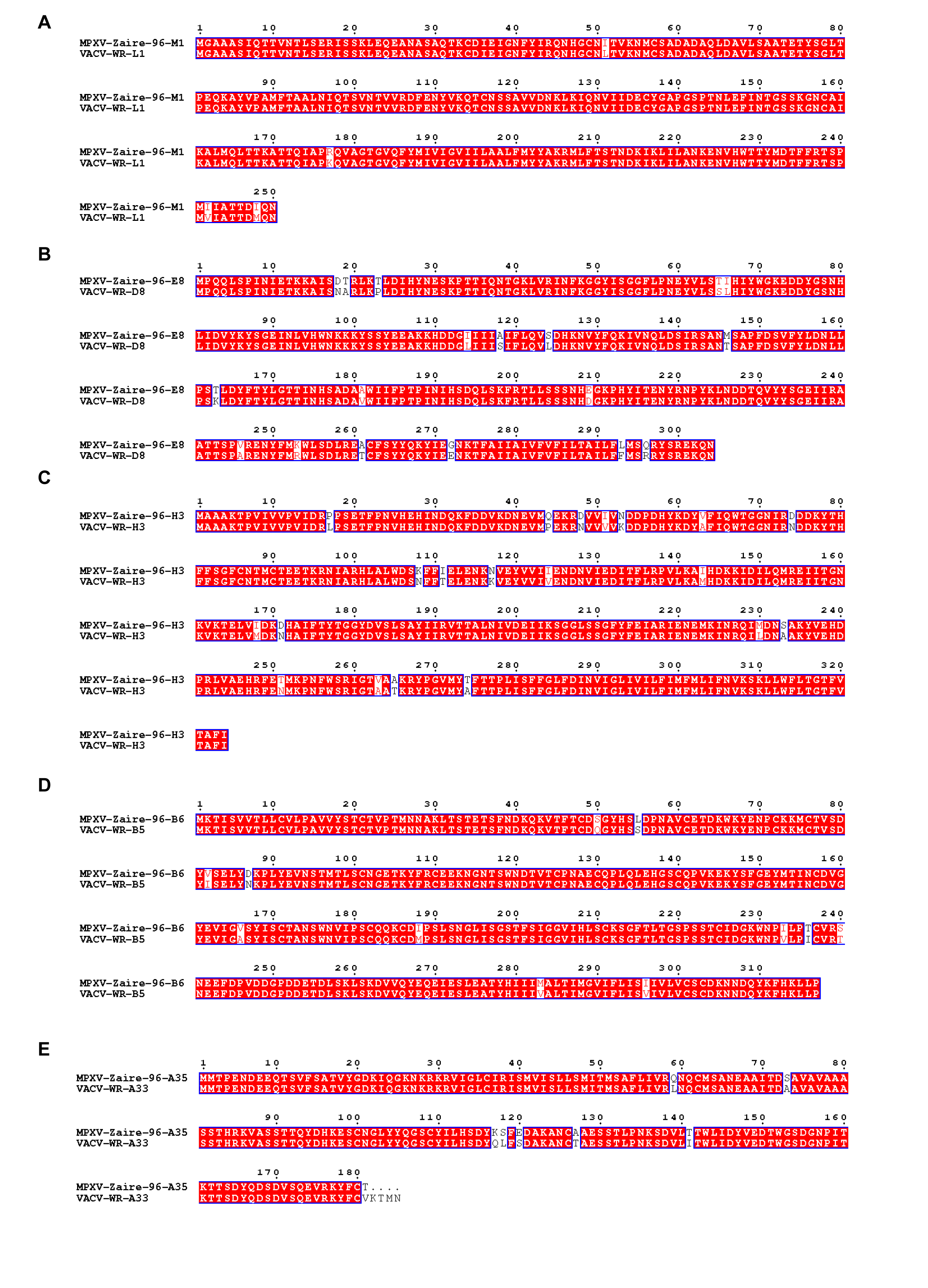

Supplement: S1 Fig — (A-E) Shown are sequence alignments of mpox clade I virus (MPXV-Zaire-96) strain proteins against orthologous proteins from vaccinia virus strain WR (VACV-WR) wild type for M1 to L1 (A), E8 to D8 (B), H3 to H3 (C), B6 to B5 (D), and A35 to A33 (E). And Amino acid identity is 98.4% for M1/L1, 94.07% for E8/D8, 93.83% for H3/H3, 96.53% for B6/B5, and 93.5% for A33/A35. (TIF) [file ppat.1013389.s001.tif]

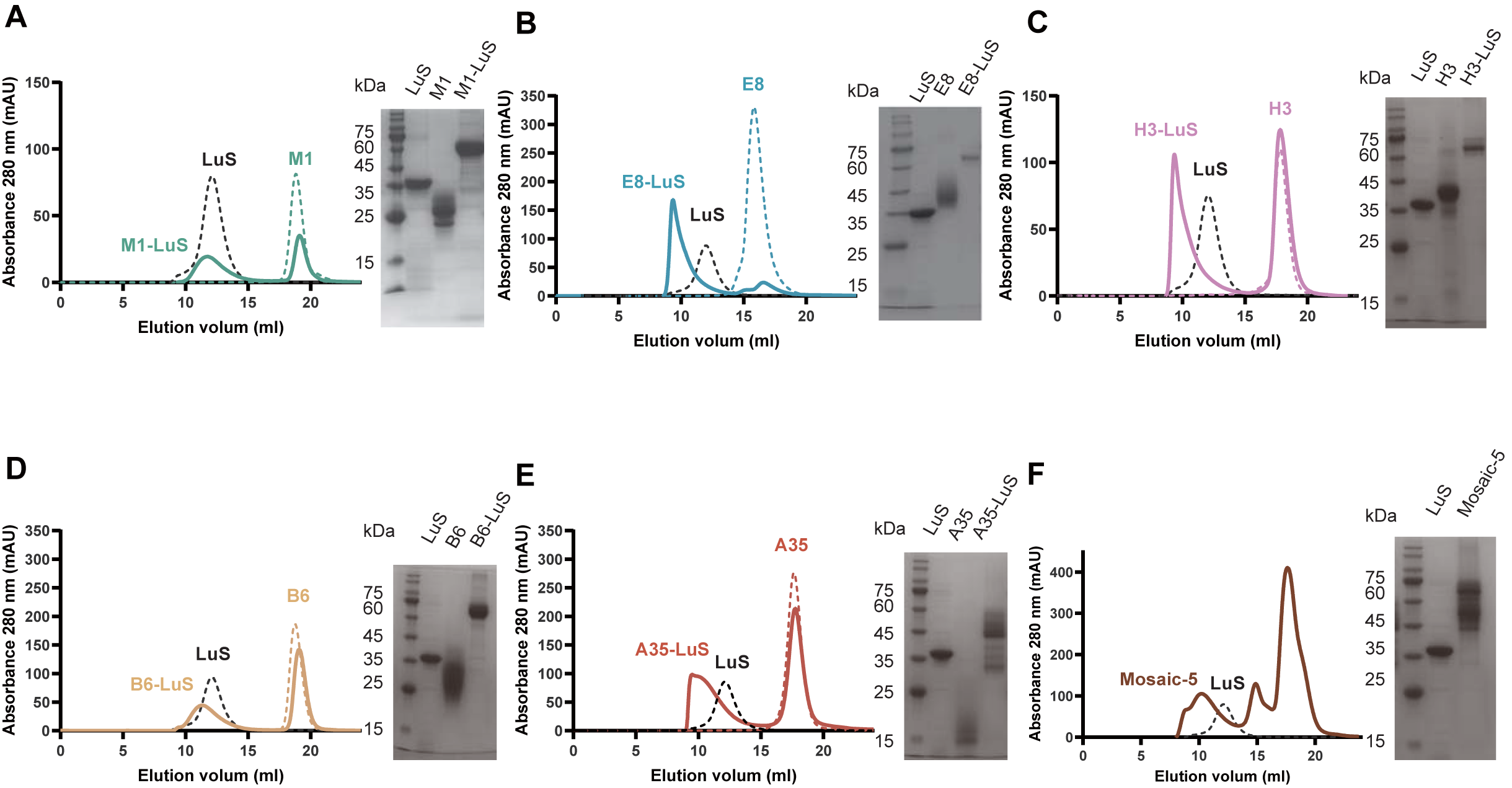

Supplement: S2 Fig — Size-exclusion chromatography trace for LuS shown via dotted black line, M1-LuS (A), E8-LuS (B), H3-LuS (C), B6-LuS (D), A35-LuS (E) and mosaic-5 (F) shown via solid color lines, and single antigen purified by HiLoad 16/600 Superdex 200 pg shown via corresponding color dotted line as control. Each antigen-LuS nanoparticle exhibited peak forward shifts of retention. The reducing SDS-PAGE analysis for the purified antigens, LuS, M1-LuS (A), E8-LuS (B), H3-LuS (C), B6-LuS (D), A35-LuS (E) nanoparticles, and each individual antigen. (TIF) [file ppat.1013389.s002.tif]

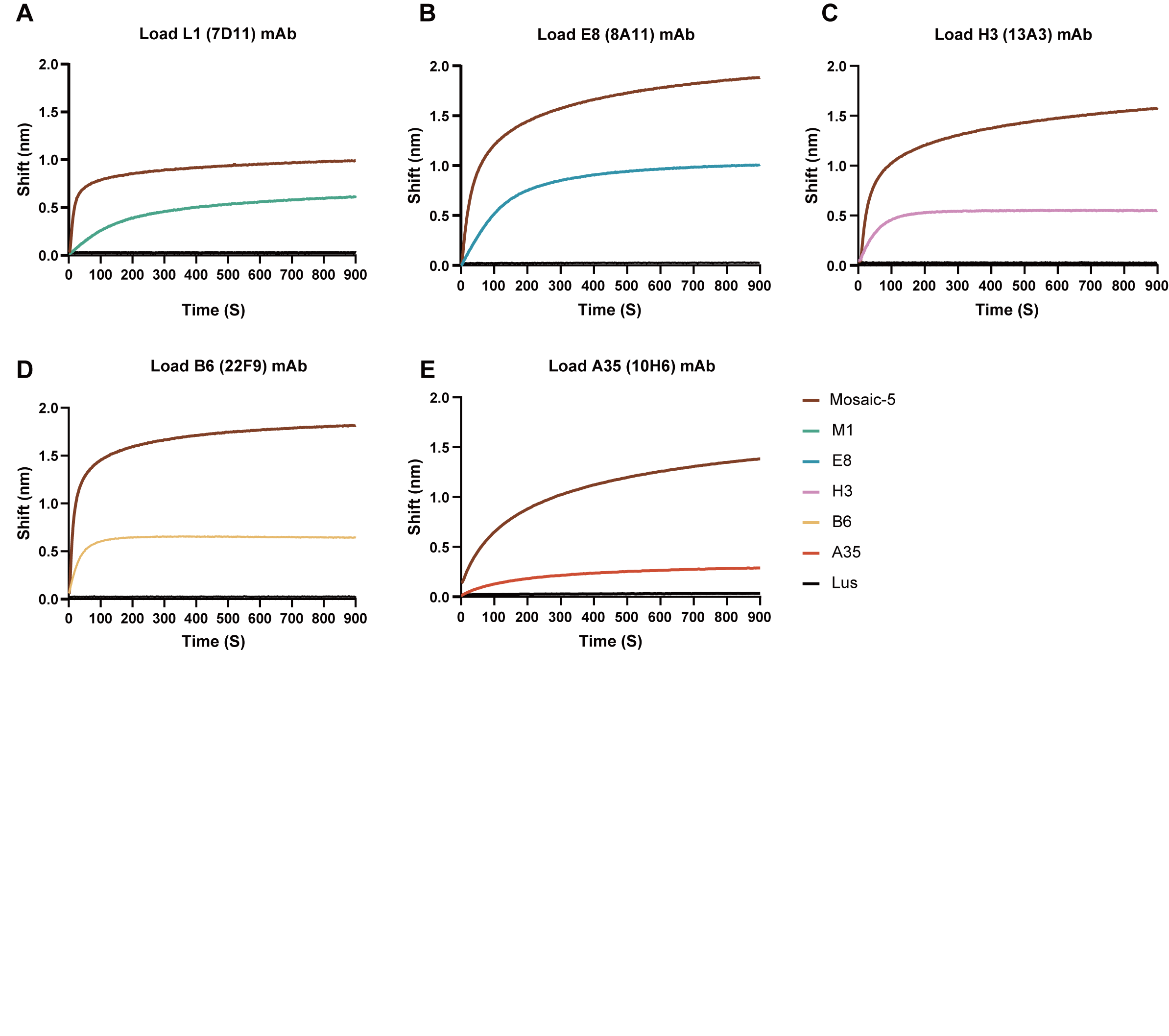

Supplement: S3 Fig — Nanoparticle immunogens and each antigen were associated with VACV L1-specific neutralizing mAb 7D11 for corresponding MPXV homologous antigen M1 (A) captured on AMC biosensors, and with E8 specific mAb 8A11 (B), H3 specific mAb 13A3 (C), B6 specific mAb 22F9 (D), and A35 specific mAb 10H6 (E) captured on ProA biosensors for 900 s, respectively. (TIF) [file ppat.1013389.s003.tif]

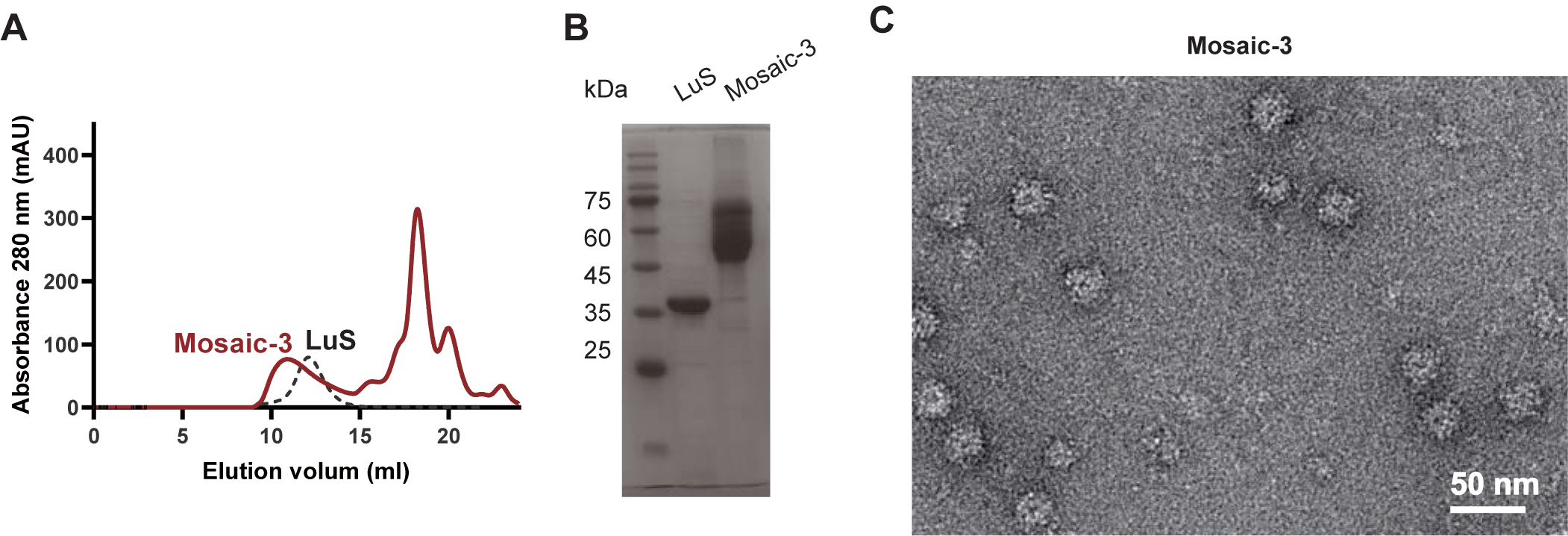

Supplement: S4 Fig — Analytical gel filtration profiles (A), SDS-PAGE analysis (B), and Negative-staining Ems (C) of mosaic were shown. Size-exclusion chromatography trace for LuS shown via dotted black line, mosaic-3 shown via solid color lines. And mosaic-3 nanoparticle exhibited peak forward shifts of retention. (TIF) [file ppat.1013389.s004.tif]

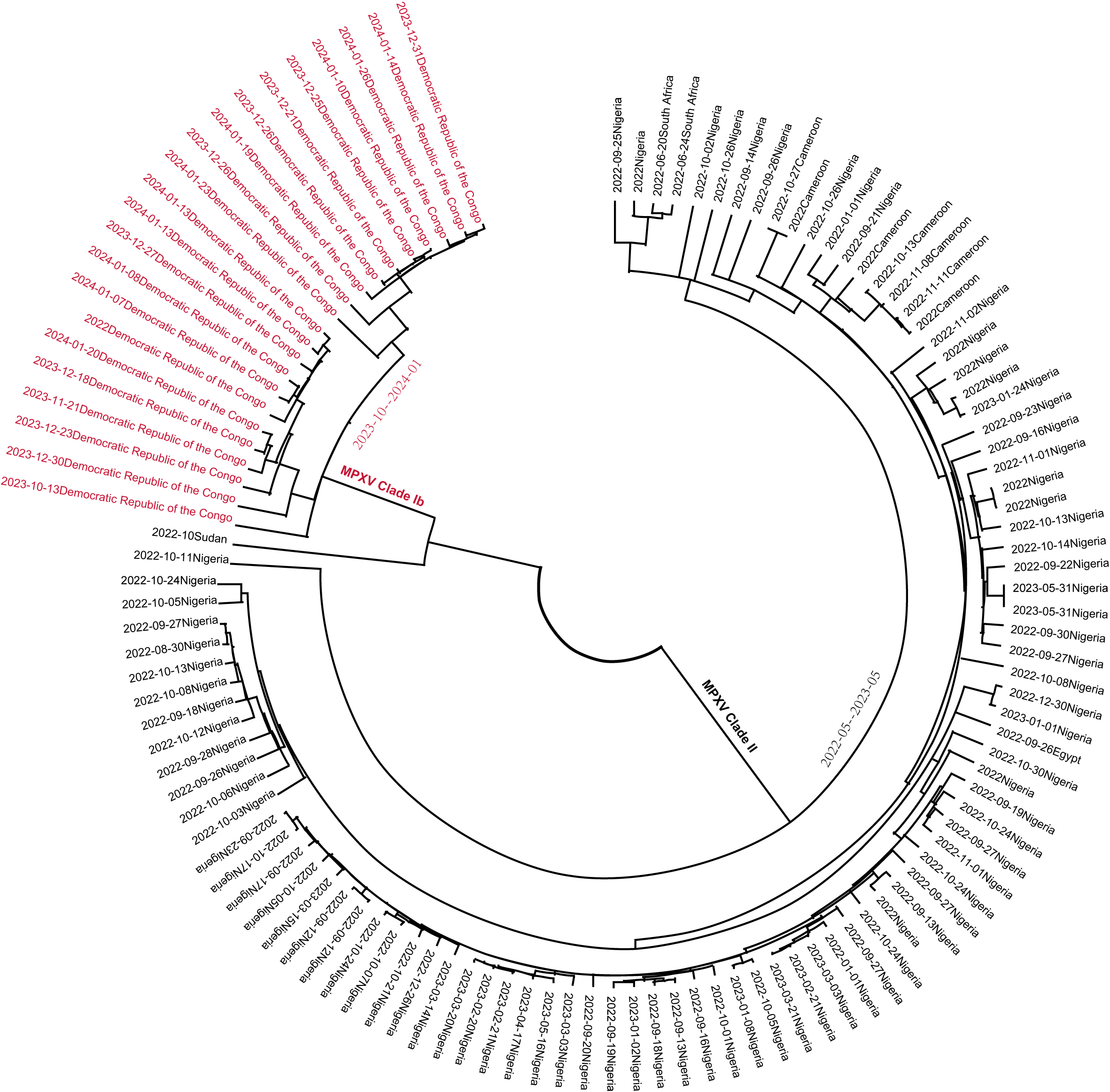

Supplement: S5 Fig — The phylogenetic tree illustrates the evolutionary relationships among various MPXV isolates collected between 2022 and 2024. The tree including 118 sequences from different geographical locations, primarily Nigeria, South Africa, Cameroon, Egypt, Sudan, and the Democratic Republic of the Congo downloaded and analysis by National Center for Biotechnology Information (NCBI). The isolates are grouped into two main clades: Clade II (Black), corresponding to the period from May 2022 to May 2023, and Clade Ib (Red), representing the interval from October 2023 to January 2024. This classification reflects distinct evolutionary lineages. (TIF) [file ppat.1013389.s005.tif]

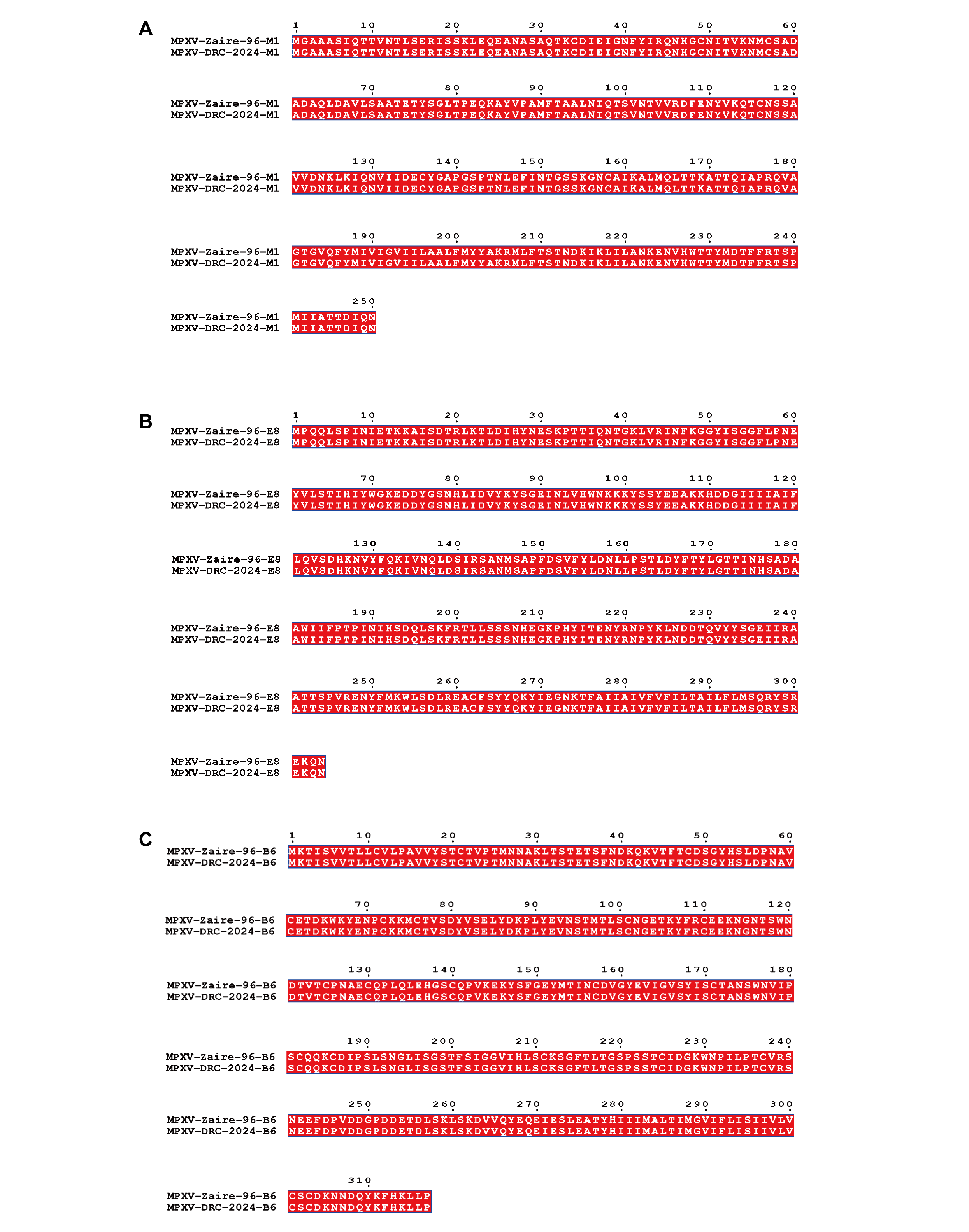

Supplement: S6 Fig — (A-C) Shown are sequence alignments between Clade I strain (MPXV-Zaire-96, accession AF380138) and Clade Ib strain (MPXV-DRC-2024, accession PP601224) for M1 (A), E8 (B) and B6 (C). The amino acid identities of three proteins are completely conserved. (TIF) [file ppat.1013389.s006.tif]
